# Structural basis of flagellar motility regulation by the MogR repressor and the GmaR antirepressor in *Listeria monocytogenes*

**DOI:** 10.1093/nar/gkac815

**Published:** 2022-10-26

**Authors:** So Yeon Cho, Hye-won Na, Han Byeol Oh, Yun Mi Kwak, Wan Seok Song, Sun Cheol Park, Wook-Jong Jeon, Hongbaek Cho, Byung-Chul Oh, Jeongho Park, Seung Goo Kang, Geun-Shik Lee, Sung-il Yoon

**Affiliations:** Division of Biomedical Convergence, College of Biomedical Science, Kangwon National University, Chuncheon 24341, Republic of Korea; Division of Biomedical Convergence, College of Biomedical Science, Kangwon National University, Chuncheon 24341, Republic of Korea; Division of Biomedical Convergence, College of Biomedical Science, Kangwon National University, Chuncheon 24341, Republic of Korea; Division of Biomedical Convergence, College of Biomedical Science, Kangwon National University, Chuncheon 24341, Republic of Korea; Institute of Bioscience and Biotechnology, Kangwon National University, Chuncheon 24341, Republic of Korea; Division of Biomedical Convergence, College of Biomedical Science, Kangwon National University, Chuncheon 24341, Republic of Korea; Department of Biological Sciences, College of Natural Sciences, Sungkyunkwan University, Suwon 16419, Republic of Korea; Department of Biological Sciences, College of Natural Sciences, Sungkyunkwan University, Suwon 16419, Republic of Korea; Lee Gil Ya Cancer and Diabetes Institute, College of Medicine, Gachon University, Incheon 406-840, Republic of Korea; College of Veterinary Medicine, Kangwon National University, Chuncheon 24341, Republic of Korea; Division of Biomedical Convergence, College of Biomedical Science, Kangwon National University, Chuncheon 24341, Republic of Korea; College of Veterinary Medicine, Kangwon National University, Chuncheon 24341, Republic of Korea; Division of Biomedical Convergence, College of Biomedical Science, Kangwon National University, Chuncheon 24341, Republic of Korea; Institute of Bioscience and Biotechnology, Kangwon National University, Chuncheon 24341, Republic of Korea

## Abstract

The pathogenic *Listeria monocytogenes* bacterium produces the flagellum as a locomotive organelle at or below 30°C outside the host, but it halts flagellar expression at 37°C inside the human host to evade the flagellum-induced immune response. *Listeria monocytogenes* GmaR is a thermosensor protein that coordinates flagellar expression by binding the master transcriptional repressor of flagellar genes (MogR) in a temperature-responsive manner. To understand the regulatory mechanism whereby GmaR exerts the antirepression activity on flagellar expression, we performed structural and mutational analyses of the GmaR–MogR system. At or below 30°C, GmaR exists as a functional monomer and forms a circularly enclosed multidomain structure via an interdomain interaction. GmaR in this conformation recognizes MogR using the C-terminal antirepressor domain in a unique dual binding mode and mediates the antirepressor function through direct competition and spatial restraint mechanisms. Surprisingly, at 37°C, GmaR rapidly forms autologous aggregates that are deficient in MogR neutralization capabilities.

## INTRODUCTION

*Listeria monocytogenes* is a foodborne pathogenic gram-positive bacterium (1,2). Ingestion of *L. monocytogenes*-contaminated dairy products and meats causes mild gastroenteritis characterized by fever, nausea, vomiting and diarrhea (3,4). However, *L. monocytogenes* infection may progress to severe listeriosis, including septicemia and meningitis, in elderly, pregnant, neonatal and immunocompromised persons with a mortality rate up to ∼20%. The control of *L. monocytogenes* infections is difficult due to its ability to proliferate even at refrigeration temperatures.

Motility plays a key role in the survival, multiplication and spread of *L. monocytogenes* inside and outside the host (5,6). *Listeria monocytogenes* employs one of two locomotion modes depending on growth temperatures. At 30°C or lower in the environment outside the human host, *L. monocytogenes* assembles flagella using diverse flagellar proteins and migrates by rotating the flagella (7). However, at 37°C in the human host, *L. monocytogenes* does not produce flagellar proteins and instead switches to a propelling movement driven by polymerization of the host actin protein (8,9). Inhibition of flagellar protein expression at 37°C facilitates the pathogenesis of *L. monocytogenes* via the evasion of host immune surveillance, given that bacterial flagellar proteins trigger both innate and adaptive immune responses in infected humans (10,11).

**Figure 1.**
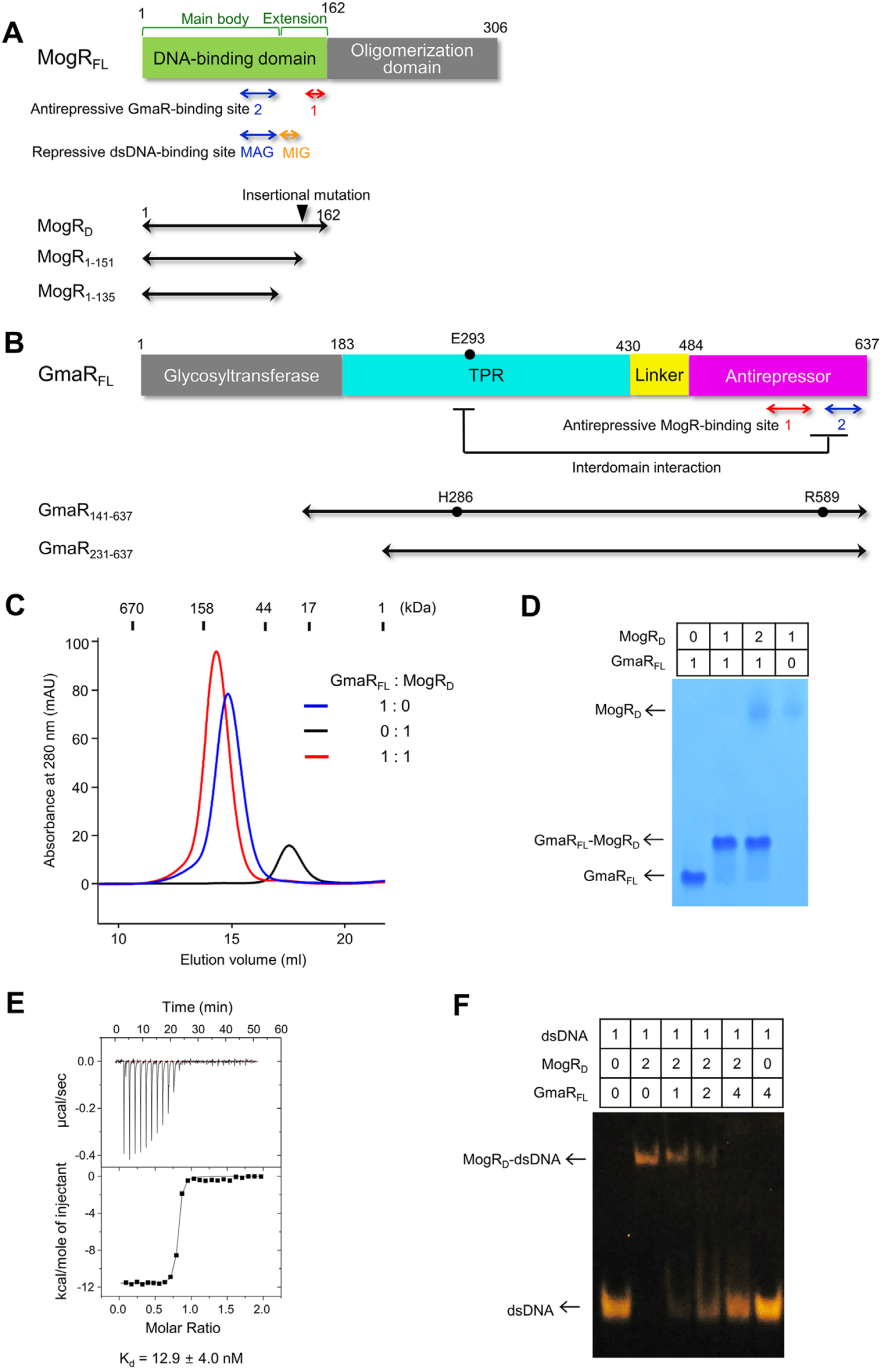
Intermolecular interaction between the GmaR antirepressor and the transcriptional repressor MogR, and the inhibitory effect of GmaR on the MogR–dsDNA interaction. (**A**) Schematic representation of the MogR proteins (MogR_FL_, MogR_D_, MogR_1-151_ and MogR_1-135_) used in this study. The antirepressive and repressive sites of MogR are indicated below the schematic diagram of MogR_FL_ that defines the MogR domains. A black triangle on MogR_D_ represents the insertional mutation site used to generate the MogR_D_^49ins^, MogR_D_^34ins^ and MogR_D_^19ins^ mutants (Figure 7B). (**B**) Schematic representation of the GmaR proteins (GmaR_FL_, GmaR_141-637_ and GmaR_231-637_) used in this study. The antirepressive sites and interdomain interaction sites of GmaR are indicated below the schematic diagram of GmaR_FL_ that defines the GmaR domains. Black dots in GmaR_FL_ and GmaR_141-637_ represent the GmaR residues (E293, H286 and R589) that were mutated to confirm the contribution of the interdomain interaction to protein thermostability. (**C–E**) GmaR–MogR complex formation. The direct interaction of GmaR_FL_ with MogR_D_ was analyzed by gel-filtration chromatography (C), native PAGE (D) and ITC (E; Wiseman *c*-value, 2068 ± 684) (42). The data are representative of three independent experiments that yielded similar results. (**F**) GmaR-mediated inhibition of the MogR_D_–dsDNA interaction in EMSA (*n* = 3 independent experiments). The molar ratio in each reaction is shown above the gel image.

In *L. monocytogenes*, flagellar expression is transcriptionally coordinated in a temperature-dependent manner by the motility gene repressor MogR and the antirepressor GmaR (12–14). *Listeria monocytogenes* MogR is the master regulator that represses the transcription of all flagellar motility genes in a nonhierarchical manner, although other motility regulators from gram-negative bacteria activate flagellar transcription in a hierarchical order (12,13,15). MogR consists of an N-terminal DNA-binding domain (DBD, the only member of the PF12181 Pfam family) and a C-terminal oligomerization domain (Figure 1A and Supplementary Figure S1) (16). When MogR binds its operator sites in the flagellar motility operons using two separate regions (major groove interaction site, MAG site; minor groove interaction site, MIG site) in the DBD, flagellar transcription is suppressed (12,16). The MogR-mediated repression of flagellar transcription can be counteracted by the antirepressor GmaR (14). GmaR forms an intermolecular complex with MogR and prevents MogR from binding to operator DNA, resulting in the derepression of flagellar transcription. GmaR-mediated derepression occurs in a temperature-dependent manner (14,17,18). At temperatures below 30°C, GmaR transcription is upregulated, and the resulting GmaR protein neutralizes MogR, acting as an antirepressor. MogR in complex with GmaR cannot function as a repressor of flagellar transcription, allowing *L. monocytogenes* to express flagellar proteins and to move through flagellum-based motility outside the host. However, when the listerial growth temperature reaches 37°C in the infected host, transcriptional restraint is applied to the *gmaR* gene to lower GmaR production. Furthermore, the GmaR protein loses its MogR-binding ability at 37°C through temperature-sensitive degradation or structural changes (18). As a result, MogR binds operator DNA and represses flagellar transcription, allowing *L. monocytogenes* to evade the host immune response. Therefore, GmaR is considered a thermosensor protein that modulates flagellar expression in response to a temperature change (18).

GmaR is a dual-function protein that consists of 637 residues (Figure 1B and Supplementary Figure S2). The N-terminal part of GmaR (∼180 residues) contains a glycosyltransferase domain that catalyzes the transfer of N-acetyl glucosamine to the flagellar filament protein flagellin (14,19). The C-terminal part of GmaR (∼460 residues) is required to exert an antagonistic effect on MogR-mediated transcriptional repression (18). Although GmaR plays a key role as a molecular thermoswitch to select the motility mode of *L. monocytogenes*, molecular information on the antirepressor GmaR is highly limited. Furthermore, due to the unavailability of structural information, it is unclear how GmaR recognizes MogR and coordinates its antirepressor function depending on temperature. To reveal the structural and molecular mechanism for the temperature-responsive regulation of flagellar expression by the MogR–GmaR system, we determined the crystal structures of GmaR alone and in complex with MogR and validated structure-based hypotheses through extensive biochemical and mutational studies.

## MATERIALS AND METHODS

### Construction of expression vectors

The *gmaR* and *mogR* genes were amplified by PCR using the genomic DNA of *L. monocytogenes* as a template to construct the full-length GmaR (GmaR_FL_; residues 1–637) and MogR (MogR_FL_; residues 1–306) expression plasmids, respectively. The resulting PCR product was digested using restriction enzymes and ligated into the pET49b plasmid modified to express the recombinant protein fused with an N-terminal hexa-histidine (His_6_) tag and a subsequent thrombin cleavage site (20). The ligation product was transformed into *Escherichia coli* DH5α cells, and a correct transformant was identified by DNA sequencing. Plasmids for recombinant expression of truncated GmaR or MogR proteins were generated similarly to the full-length protein expression plasmid except for the PCR step, in which the DNA fragment encoding the truncated protein was amplified using the full-length protein expression plasmid as a template. The point mutation of GmaR and MogR was performed based on the QuikChange site-directed mutagenesis protocol (Agilent) and confirmed by DNA sequencing.

### Protein expression and purification

The GmaR expression plasmid was transformed into *E. coli* BL21 (DE3) cells to obtain GmaR protein (GmaR_FL_, truncated GmaR and point mutant GmaR). The cells were cultured in LB medium at 37°C. When the optical density of the culture at 600 nm reached ∼0.6, GmaR protein was overexpressed at 18°C overnight in the presence of 1 mM isopropyl β-D-1-thiogalactopyranoside (IPTG). The cells containing GmaR protein were harvested by centrifugation at 4°C. The resulting cell pellet was resuspended in a lysis solution containing 50 mM Tris, pH 8.0, 200 mM NaCl, and 5 mM β-mercaptoethanol (βME) and sonicated with 1 mM phenylmethylsulfonyl fluoride (PMSF) on ice. After centrifugation at 4°C, the supernatant was incubated with Ni-NTA resin (Qiagen) in the presence of 10 mM imidazole at 4°C for 1 h. The resin was packed into an Econo-Column (Bio-Rad) and washed with lysis solution containing 10 mM imidazole at room temperature. GmaR protein was eluted from the resin at room temperature in a stepwise manner using 50, 100 and 250 mM imidazole and dialyzed at 4°C against 20 mM Tris, pH 8.0, 150 mM NaCl and 5 mM βME. The resulting GmaR protein was treated with thrombin at 18°C to remove the N-terminal His_6_ tag and further purified by gel-filtration chromatography at room temperature using a Superdex 200 HR 16/600 column (GE Healthcare) in 20 mM Tris, pH 8.0, 150 mM NaCl and 5 mM βME.

The GmaR_141-637_^H286C/R589C^ protein was obtained via overexpression in the *E. coli* strain SHuffle and first purified by Ni-NTA affinity chromatography without βME. The His_6_-tagged GmaR_141-637_^H286C/R589C^ protein was further purified by gel-filtration chromatography in the absence of βME without thrombin treatment. For a comparative analysis of protein thermostability, the His_6_-tagged GmaR_141-637_ protein was also obtained in an identical manner to GmaR_141-637_^H286C/R589C^.

MogR protein (MogR_FL_, truncated MogR and point mutant MogR) was overexpressed in *E. coli* BL21 (DE3) cells and purified by affinity chromatography using Ni-NTA resin with a similar procedure to GmaR protein. MogR protein was further purified by ion-exchange chromatography using Mono S 10/100 or Mono Q 10/100 columns (GE Healthcare) with a NaCl gradient of 0–500 mM. The purified MogR protein was dialyzed against 20 mM Hepes, pH 7.4, 150 mM NaCl and 5 mM βME.

To obtain selenomethionine (SeMet)-labeled GmaR_141-637_ protein (SeMet-GmaR_141-637_), the GmaR_141-637_ expression plasmid was transformed into the *E. coli* strain B834 (DE3), and the transformed cells were grown in nutrient-supplemented M9 minimal medium (Molecular Dimensions) containing 40 μg/ml L-SeMet. The SeMet-GmaR_141-637_ protein was purified by Ni-NTA affinity chromatography and gel-filtration chromatography with the identical procedure used for the native GmaR protein.

To generate the GmaR–MogR complex for crystallization, an N-terminally truncated GmaR protein (GmaR_231-637_; residues 231–637) and the MogR DBD protein (MogR_D_; residues 1–162) were individually overexpressed in *E. coli* BL21 (DE3) cells as described above. The cells containing the GmaR_231-637_ protein were mixed with the MogR_D_ counterparts, and the mixture was sonicated in lysis solution containing 1 mM PMSF. During this lysis step, the GmaR_231-637_ protein forms a complex with the MogR_D_ protein. The GmaR_231-637_–MogR_D_ complex was initially purified by Ni-NTA affinity chromatography and then treated with thrombin. Further purification was performed by anion-exchange chromatography using a Mono Q 10/100 column (GE Healthcare).

### Crystallization and X-ray diffraction

For crystallization, the GmaR_141-637_ protein and the GmaR_231-637_–MogR_D_ complex were concentrated to over 10 mg/ml at 4°C. Crystallization was carried out at 18°C by a sitting-drop vapor-diffusion method using a drop consisting of 0.5 μl of protein and 0.5 μl of reservoir solution. GmaR_141-637_ crystals were obtained using a reservoir solution containing 0.9–1.5 M sodium acetate and 0.1 M sodium cacodylate, pH 6.5–7.4, and cryoprotected by 25% ethylene glycol. The GmaR_231-637_–MogR_D_ complex was crystallized using a reservoir solution containing 25.5% PEG 4000, 0.17 M ammonium acetate, 15% glycerol and 0.085 M sodium citrate, pH 5.6. The crystals were flash-cooled under a nitrogen cryostream at −173°C. X-ray diffraction was performed at beamline 7A at the Pohang Accelerator Laboratory (South Korea). X-ray diffraction data were processed using the HKL2000 program (21). Data collection statistics are listed in Supplementary Tables S1 and S2.

### Structure determination

The GmaR_141-637_ structure was determined by single-wavelength anomalous diffraction (SAD) phasing with the Phenix AutoSol program using X-ray diffraction data obtained from the crystal of the SeMet-GmaR_141-637_ protein (22). The anomalous signal extended to 2.95 Å resolution based on anomalous measurability (>0.05) in the AutoSol program. The GmaR_231-637_–MogR_D_ complex structure was determined by molecular replacement using the GmaR_141-637_ structure and the MogR_D_ structure (PDB ID: 3FDQ) as search models (16,23). The GmaR_141-637_ and GmaR_231-637_–MogR_D_ models were refined using the Phenix program and manually modified using the Coot program (24,25). The final GmaR_141-637_ and GmaR_231-637_–MogR_D_ structures exhibited good stereochemistry without outliers in the Ramachandran plot. Refinement statistics are listed in Supplementary Table S2.

### Native PAGE

The MogR-binding capacity and protein thermostability of GmaR were addressed by native PAGE. To assess the MogR-GmaR interaction, GmaR protein was incubated with MogR protein at a defined molar ratio at 18°C for 30 min in an interaction solution containing 20 mM Hepes, pH 7.4, 150 mM NaCl and 5 mM βME. To analyze the thermostability of GmaR, GmaR protein was incubated at different temperatures for a defined time using water bath equipment. To evaluate the MogR-binding activity of the temperature-sensitive GmaR protein, GmaR protein was pretreated at different temperatures and subsequently incubated with MogR at 18°C in the interaction solution or βME-free interaction solution. The sample was loaded on a native 6% polyacrylamide gel and subjected to electrophoresis for 150 min at 100 V at pH 8.8. Protein bands in the gel were visualized with Coomassie brilliant blue.

### Gel-filtration chromatography

The direct interaction of GmaR_FL_ or GmaR_231-637_ with MogR_D_ was analyzed by gel-filtration chromatography at room temperature using an elution solution containing 20 mM Hepes, pH 7.4, 150 mM NaCl and 5 mM βME. Protein samples (385 μg GmaR_FL_ or 248 μg GmaR_231-637_; 100 μg MogR_D_; their mixture at a 1:1 molar ratio) in 300 μl of the elution solution were loaded onto a Superdex 200 10/300 column. Protein elution was monitored by measuring the absorbance at 280 nm.

The thermostability and temperature-dependent MogR_D_ binding of GmaR were also analyzed by gel-filtration chromatography. To assess the thermostability of GmaR_FL_, the GmaR_FL_ protein (192 μg) was incubated at 30°C or 37°C for 5 min or 15 min and then loaded onto a Superdex 200 10/300 column. To assess the MogR_D_-binding activity of temperature-sensitive GmaR protein, the GmaR_FL_ protein (192 μg) was preincubated at 37°C for 15 min and then incubated with MogR_D_ (50 μg) at a 1:1 molar ratio at 18°C for 30 min. The 1:1 mixture or its individual components were loaded onto a Superdex 200 10/300 column. Gel-filtration chromatography was performed at room temperature in the elution solution. Protein elution was monitored by measuring the absorbance at 280 nm. The elution fractions were analyzed by SDS-PAGE.

### Isothermal titration calorimetry

The interaction of GmaR_FL_ or GmaR_141-637_ with MogR_D_ was quantitively analyzed by isothermal titration calorimetry (ITC). ITC was performed using a MicroCal iTC200 instrument (Malvern) at 18°C with a stirring speed of 750 rpm. Protein samples were prepared by simultaneously dialyzing the GmaR (GmaR_FL_ or GmaR_141-637_) and MogR_D_ proteins at 4°C against 20 mM Hepes, pH 7.4, 150 mM NaCl and 5 mM βME. For calorimetric titration, 250 μM MogR_D_ was injected multiple times into a sample cell containing 25 μM GmaR_FL_ or GmaR_141-637_. ITC data were evaluated with the Origin 7 program (MicroCal) using the one-site binding model.

### Enzyme-linked immunosorbent assay

An enzyme-linked immunosorbent assay (ELISA) was performed to quantitatively address the interaction of the GmaR_FL_ protein with truncated or point mutant proteins of MogR. Each well in a 96-well plate was coated with MogR protein (20 nM) or a negative control protein (FliD, 20 nM) in phosphate-buffered saline at 4°C overnight and was incubated for 2 h at room temperature with a blocking solution containing phosphate-buffered saline with 0.05% Tween 20 (PBST) and 1% bovine serum albumin. Three-fold serially diluted His_6_-tagged GmaR_FL_ protein in the blocking solution was added to the wells and incubated for 2 h at 18°C. After washes with the PBST solution, the well was incubated serially with mouse anti-His_6_ antibody, horseradish peroxidase-conjugated goat anti-mouse antibody and 3,3',5,5'-tetramethylbenzidine substrate. The horseradish peroxidase reaction was stopped using sulfuric acid, and the absorbance at 450 nm was measured using an Epoch microplate spectrophotometer (BioTek).

ELISA was also employed to assess the inhibitory effect of the operator dsDNA on the interaction between GmaR_FL_ and MogR_1-135_. Each well in a 96-well plate was coated with MogR_1-135_ protein (100 nM) or the negative control protein FliD (100 nM) and then blocked for 2 h at room temperature with the blocking solution. The plate was further incubated for 2 h at 18°C with the His_6_-tagged GmaR_FL_ protein (5 μM) in the presence or absence of the operator dsDNA (100 μM; 5'-A_1_TTTGATTTTTTAAAAAAATGAAGA_25_-3'). The subsequent procedures are identical to those of the ELISA that was used to address the interaction of GmaR_FL_ with truncated or point mutant proteins of MogR.

### Fluorescence polarization assay

A fluorescence polarization (FP) assay was performed to determine the dsDNA-binding affinity of MogR and to address GmaR-mediated inhibition of the MogR–dsDNA interaction. dsDNA was generated by incubating a fluorescein-labeled oligonucleotide (5'-A_1_TTTGATTTTTTAAAAAAATGAAGA_25_-3') and its unlabeled complementary counterpart (5'-T_1_CTTCATTTTTTTAAAAAATCAAAT_25_-3') at 95°C for 15 min and then slowly cooling the reaction to room temperature. For the MogR–dsDNA interaction assay, the resulting dsDNA (1 nM) was incubated with 3-fold serially diluted MogR protein in 20 mM Hepes, pH 7.4, 150 mM NaCl and 5 mM βME. To assess the inhibitory effect of GmaR on the MogR–dsDNA interaction, 3-fold serially diluted GmaR_FL_ protein (0.5–10 μM) was added to the mixture of dsDNA (1 nM) and MogR (100 nM). FP signals were measured at room temperature using an Infinite F200 PRO instrument (Tecan; excitation wavelength, 485 nm; emission wavelength, 535 nm) and analyzed with the one-site binding or dose–response inhibition model using the Prism 5 program (GraphPad).

### Electrophoretic mobility shift assay

To analyze the MogR–dsDNA interaction and the inhibitory activity of GmaR on the MogR–dsDNA interaction, an electrophoretic mobility shift assay (EMSA) was performed using MogR protein and the operator dsDNA (5'-A_1_TTTGATTTTTTAAAAAAATGAAGA_25_-3') in the presence or absence of GmaR_FL_. For the interaction assay, the purified MogR protein was incubated with dsDNA at a defined molar ratio at 18°C for 30 min in a solution containing 20 mM Hepes, pH 7.4, 150 mM NaCl and 5 mM βME. For the GmaR-mediated inhibition assay, dsDNA was incubated with MogR protein at a 1:2 molar ratio at room temperature for 10 min. Next, the MogR–dsDNA mixture was incubated with the GmaR_FL_ protein at a defined molar ratio at room temperature for 10 min. Samples were electrophoretically separated on an 8% polyacrylamide gel in a Tris-borate-EDTA solution for 75 min at 100 V. DNA bands were visualized by ethidium bromide staining.

The inhibitory activity of temperature-sensitive GmaR on the MogR–dsDNA interaction was also analyzed by EMSA. GmaR protein was preincubated at different temperatures and then incubated with the 2:1 MogR_D_:dsDNA mixture at room temperature for 10 min. The samples were subjected to EMSA.

### Pull-down assay

For a pull-down assay, a dual expression vector that was designed to coexpress the MogR_D_ (or its mutants) and His_6_-tagged GmaR_FL_ proteins was generated by ligating MogR and GmaR_FL_-encoding DNA fragments into the pETDuet-1 plasmid (Novagen). The MogR and His_6_-tagged GmaR_FL_ proteins were coexpressed in *E. coli* BL21 (DE3) cells at 18°C overnight in the presence of IPTG. The His_6_-tagged GmaR_FL_ protein was purified by affinity chromatography using Ni-NTA resin in 50 mM Tris, pH 8.0, 100 mM NaCl and 5 mM βME. The MogR protein that was copurified with GmaR_FL_ was analyzed by SDS-PAGE and Coomassie brilliant blue staining.

### *Escherichia coli* two-hybrid interaction assay

A bacterial adenylate cyclase-based two-hybrid system was used to verify the temperature-sensitive association of GmaR with MogR and to reveal the critical roles of the site-1 and interdomain interactions in GmaR–MogR binding (26,27). For the two-hybrid interaction assay in *E. coli*, the pCH363 and pKNT25 plasmids were genetically engineered to express the GmaR_FL_ (or its mutant) and MogR_D_ (or its mutant) proteins fused with the complementary T18 and T25 fragments of adenylate cyclase, respectively (28). The resulting pCH363 and pKNT25 plasmids were cotransformed into *E. coli* BTH101 reporter cells. The transformant cells were cultured at 30°C or 37°C in LB medium containing 0.5 mM IPTG for protein expression until the absorbance of the culture at 600 nm (*A*_600_) reached 0.3–0.4. To determine the β-galactosidase activity of the cells, the cultured cells were lysed using PopCulture reagent (Novagen) and lysozyme for 10 min and then treated with the 2-nitrophenyl β-D-galactopyranoside substrate in Z-buffer containing 60 mM Na_2_HPO_4_, 40 mM NaH_2_PO_4_, 10 mM KCl, 1 mM MgSO_4_ and 50 mM βME. The β-galactosidase reaction was stopped using sodium carbonate, and the absorbances at 420 nm and 550 nm (*A*_420_ and *A*_550_, respectively) were measured using an Epoch microplate spectrophotometer (BioTek). β-Galactosidase activity was determined in Miller units based on the equation [(*A*_420_ − 1.75 × *A*_550_) × 1000] / [*A*_600_ × reaction time (min) × volume of culture used in the assay (ml)].

## RESULTS

### MogR-binding and MogR-counteracting activities of GmaR

GmaR has been reported to interfere with the interaction between MogR and its operator DNA by recognizing MogR (14). To address the MogR-neutralizing activity of GmaR at a molecular level, we individually produced the GmaR and MogR proteins using an *E. coli* expression system. The full-length protein of GmaR (GmaR_FL_) was purified as a soluble monomer based on gel-filtration chromatography (Figure 1C). However, the full-length MogR protein (MogR_FL_) has been reported to form a high-order oligomer through the oligomerization domain (16). To avoid any unwanted analytical complexity caused by the high oligomeric state of MogR_FL_, a truncated MogR protein corresponding to the N-terminal DBD (MogR_D_; residues 1–162) was used throughout this study (16). As observed with GmaR_FL_, the MogR_D_ protein was monomeric in solution (Figure 1C).

The direct intermolecular interaction between GmaR_FL_ and MogR_D_ was characterized by various biophysicochemical methods, including gel-filtration chromatography, native PAGE and isothermal titration calorimetry (ITC) (Figure 1C–E). In the gel-filtration chromatography analysis, a 1:1 molar mixture of GmaR_FL_ and MogR_D_ only generated one peak corresponding to the molecular size of the GmaR_FL_–MogR_D_ complex, indicating that GmaR_FL_ directly interacts with MogR_D_ with a binding stoichiometry of 1:1 (Figure 1C). The observed 1:1 stoichiometry was verified by native PAGE, in which GmaR_FL_ completely changed its mobility in the presence of MogR_D_ at a 1:1 molar ratio (Figure 1D). In addition, the MogR-binding affinity of GmaR was quantitatively determined using ITC with a dissociation equilibrium constant (*K*_d_) of ∼13 nM, demonstrating that GmaR potently interacts with MogR (Figure 1E). Consistent with the ability of GmaR_FL_ to bind MogR_D_, the GmaR_FL_ protein interfered with the interaction between MogR_D_ and the operator dsDNA in a dose-dependent manner in an electrophoretic mobility shift assay (EMSA) (Figure 1F).

### Interdomain interaction-mediated circular structure of GmaR

**Figure 2.**
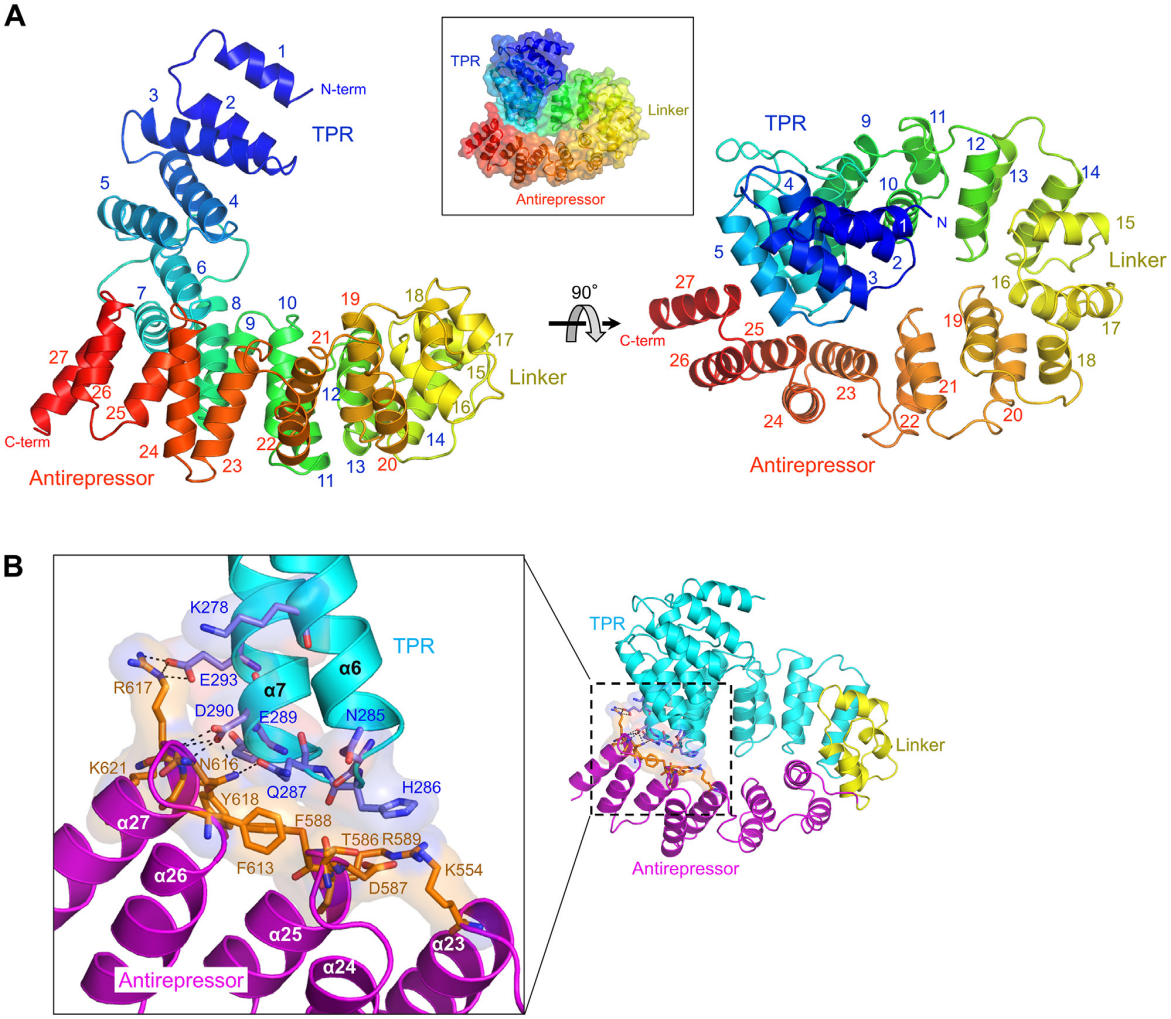
GmaR^Apo^ structure and its interdomain contacts. (**A**) Crystal structure of GmaR^Apo^ containing the TPR, linker and antirepressor domains. The GmaR^Apo^ structure is shown as rainbow ribbons [N-terminus (N-term), blue; C-terminus (C-term), red]. The α-helices of GmaR^Apo^ are numbered in domain-specific colors (TPR, blue; linker, yellow; antirepressor, red). The GmaR^Apo^ structure is also depicted in a box as rainbow ribbons with transparent surfaces in a 55°-rotation view of the left figure to highlight the circularly enclosed conformation of GmaR. (**B**) Interdomain contacts of GmaR^Apo^ between the TPR and antirepressor domains. Interdomain interface residues in the TPR (cyan ribbons) and antirepressor (magenta ribbons) domains are shown as light blue and orange sticks, respectively, with transparent surfaces. Interdomain hydrogen bonds are represented by dashed lines.

The antirepressor GmaR was structurally characterized as the first step toward understanding the GmaR-mediated derepression mechanism. Based on the expression, purification, crystallization and X-ray diffraction screens of various GmaR constructs, we determined the crystal structure of an N-terminally truncated GmaR protein (GmaR_141-637_; residues 141–637), which contains GmaR residues 183–630 in the C-terminal part without the N-terminal glycosyltransferase domain (Figure 2A and Supplementary Figure S2). GmaR_141-637_ exhibited a similar MogR_D_-binding affinity (∼22 nM) to that of GmaR_FL_ in ITC (Supplementary Figure S3).

The GmaR_141-637_ protein in the apo form (GmaR^Apo^) adopts an all α-helical structure with 27 α-helices (α1–α27) that are laterally stacked from the N-terminal α1 helix to the C-terminal α27 helix into a curved shape (Figure 2A and Supplementary Figure S4). The GmaR^Apo^ structure can be defined as a repetitive assembly of helix-turn-helix (HTH) motifs, in each of which the first and second α-helices are positioned on the concave and convex sides of the curved structure, respectively. GmaR^Apo^ is segmented into three domains (TPR, linker and antirepressor domains in order from the N-terminus to the C-terminus) and folds into a tertiary structure that resembles a duck swim ring. The N-terminal one-third of the TPR domain corresponding to the α1-α6 helices forms the vertically protruding head of the duck swim ring-like structure, and the remaining C-terminal part of GmaR^Apo^ corresponding to the α7-α27 helices assembles into a circle at the base of this structure.

The N-terminal TPR domain of GmaR^Apo^ forms a twisted semicircle using 14 α-helices (α1–α14) (Supplementary Figures S2 and S4A). The TPR domain contains four tetratricopeptide repeats (TPRs; α2-α3, α4-α5, α6-α7 and α8-α9 helices), each of which adopts the canonical HTH structure of the TPR (Supplementary Figures S2, S4A and S5A) (29,30). The two following helix pairs (α10-α11 and α12-α13 helices) in the TPR domain lack the conserved pattern of the TPR motif but fold into a typical HTH structure similar to those of the TPRs. The TPR domain is connected to the antirepressor domain via a four-helix bundle structure (α15-α18) in the linker domain (Figure 2A; Supplementary Figures S2 and S4B). The antirepressor domain consists of 9 antiparallel α-helices (α19-α27) and forms a curved HTH repeat structure (Figure 2A; Supplementary Figures S2, S4C and S5B). In the GmaR^Apo^ structure, the curved TPR and antirepressor domains are arranged in an almost perpendicular orientation (Figure 2A).

The circularly enclosed conformation of GmaR^Apo^ is stabilized through an intramolecular interdomain interaction between the TPR and antirepressor domains (Figure 2B). GmaR residues in the middle of the TPR domain (α6 and α7 helices and α6-α7 loop) interact with residues from the C-terminal region of the antirepressor domain (α25 and α27 helices and α24-α25 and α26-α27 loops). The interdomain interaction is primarily hydrophilic with eight hydrogen bonds and salt bridges. Hydrophobic residues are also observed, but only on the antirepressor domain side, and compose one-third of the interdomain interface in the antirepressor domain.

### Interaction of GmaR with MogR

**Figure 3.**
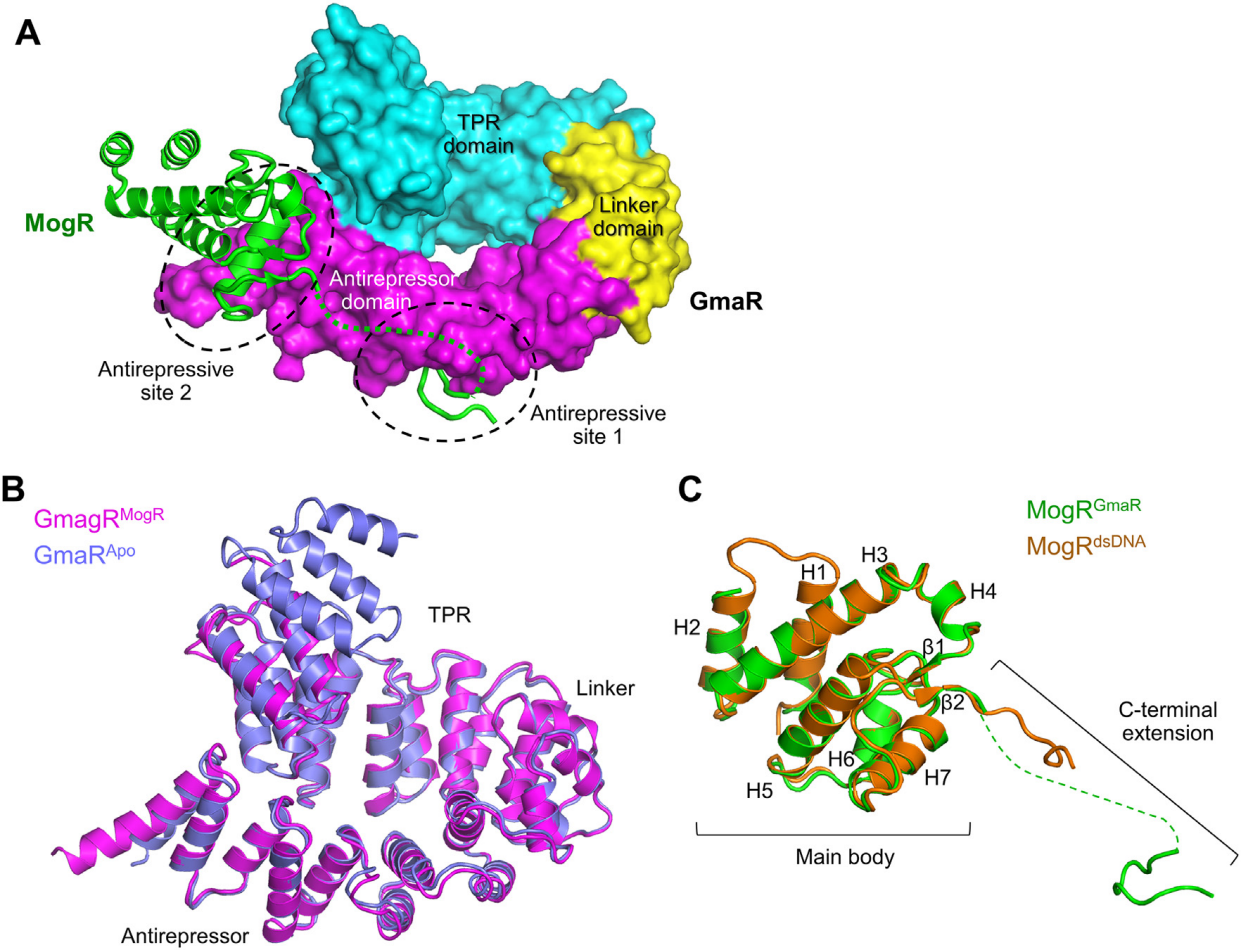
Crystal structure of the GmaR-MogR complex. (**A**) Overall structure of the GmaR–MogR complex. GmaR is depicted as a surface representation in domain-specific colors (TPR, cyan; linker, yellow; antirepressor, magenta). MogR is shown as green ribbons, and its disordered region (residues 137–151) is represented by green dotted lines. Two distinct antirepressive sites (sites 1 and 2) that correspond to the intermolecular interfaces between GmaR and MogR are highlighted by black dashed circles. The orientation of the figure is identical to that of the inset of Figure 2A. (**B**) Structural similarity of GmaR irrespective of MogR binding. The GmaR^MogR^ structure (magenta ribbons) is overlaid on the GmaR^Apo^ structure (light blue ribbons). (**C**) Structural comparison of MogR^GmaR^ with MogR^dsDNA^. The main body of MogR exhibits similar structures irrespective of binding partners. However, the C-terminal extension of MogR adopts different structures depending on binding partners. The MogR^GmaR^ structure (green ribbons) is overlaid on the MogR^dsDNA^ structure (orange ribbons; PDB ID: 3FDQ).

To visualize the GmaR–MogR interaction that is required for derepression of flagellar expression, a GmaR–MogR complex structure was determined by X-ray crystallography using the MogR_D_ protein and an N-terminally truncated GmaR protein (GmaR_231-637_) that contains residues 231–637 (Figure 3A). In the GmaR–MogR complex, one polypeptide chain of GmaR interacts with one MogR chain, consistent with the 1:1 molar ratio observed by native PAGE and gel-filtration chromatography. The asymmetric unit of the GmaR–MogR crystal contains three copies of the 1:1 complex, which exhibit similar quaternary structures (Supplementary Figures S6 and S7). MogR-bound GmaR (GmaR^MogR^) adopts a duck swim ring-like circular structure consisting of three α-helical domains, similar to the GmaR^Apo^ structure, with a root-mean-square deviation value of 1.27 Å (393 Cα atoms) (Figure 3B). In GmaR-bound MogR (MogR^GmaR^), seven α-helices and one two-stranded β-sheet are combined into a single globular structure forming the main body of MogR (Figure 3A and C; Supplementary Figure S1). The main body (MogR residues 5–136) discontinuously extends to a C-terminal 10-residue coil structure (residues 152–161) via a disordered in-between region (residues 137–151). The main body of MogR^GmaR^ has a structure similar to that of dsDNA-bound MogR_D_ (MogR^dsDNA^), but the C-terminal extension differs. The MogR^dsDNA^ structure is built up to residue 143 and consists of the main body (residues 4–136) and its continuously connected C-terminal tail (residues 137–143) without residues 144–162 (16).

**Figure 4.**
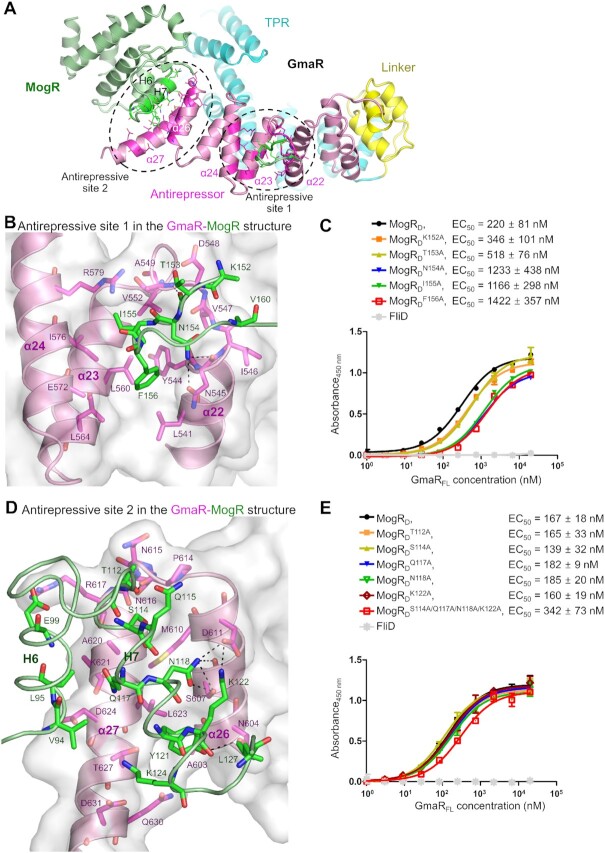
Intermolecular binding sites of GmaR and MogR. (**A**) Intermolecular interactions in the GmaR–MogR structure. GmaR is shown as ribbons in domain-specific colors (TPR, cyan; linker, yellow; antirepressor, pink). MogR is represented by light green ribbons. The intermolecular interface residues of GmaR and MogR are shown as magenta and green lines, respectively. Antirepressive sites 1 and 2 are highlighted by black dashed circles. The orientation of the figure is identical to that of Figure 3A. (**B**) Antirepressive site 1. The MogR residues at site 1 are depicted as green sticks in the C-terminal extension of MogR (light green coil). The GmaR residues at site 1 are shown as magenta sticks on the GmaR^MogR^ structure (pink ribbons and gray surfaces). Intermolecular hydrogen bonds are represented by black dashed lines. (**C**) Contributions of site-1 residues to GmaR–MogR binding based on the mutational analysis. MogR residues at site 1 (K152, T153, N154, I155 and F156) were individually mutated to alanine in MogR_D_, and the GmaR_FL_-binding capacities of the MogR_D_ mutants were analyzed by ELISA. The FliD protein was used as a negative control. The data are representative of three independent experiments that yielded similar results, and the EC_50_ values represent the means ± S.D. from the three independent experiments. (**D**) Antirepressive site 2. The MogR and GmaR residues at site 2 are depicted as green and magenta sticks in the MogR (light green coils) and GmaR (pink ribbons and gray surfaces) structures, respectively. Intermolecular hydrogen bonds are represented by dashed lines. (**E**) Contributions of site-2 residues to GmaR–MogR binding based on the mutational analysis. ELISA was employed to determine the GmaR_FL_-binding capacities of the site-2 mutants of MogR_D_ (MogR_D_^T112A^, MogR_D_^S114A^, MogR_D_^Q117A^, MogR_D_^N118A^, MogR_D_^K122A^ and MogR_D_^S114A/Q117A/N118A/K122A^). The data are representative of three independent experiments that yielded similar results, and the EC_50_ values represent the means ± S.D. from the three independent experiments.

In the GmaR–MogR complex structure, the intermolecular interaction between GmaR and MogR occurs in two spatially separated regions, antirepressive sites 1 and 2 (buried surface areas of ∼350 and ∼540 Å^2^, respectively) (Figure 4A). Antirepressive sites 1 and 2 are observed in the C-terminal extension and main body of MogR, respectively. At antirepressive site 1, a small cavity in GmaR on the convex side in the middle of the antirepressor domain accommodates MogR residues 152–156 and 160 from the C-terminal coil structure (Figure 4A and B; Supplementary Figure S8). The GmaR cavity is mainly generated by hydrophobic residues from the α22, α23 and α24 helices. The hydrophobic side chains of the MogR I155 and F156 residues at the protrusion of the C-terminal coil structure are embedded into the hydrophobic cavity of GmaR. Moreover, the N154 residue of MogR forms multiple hydrogen bonds with the GmaR residue N545. An ELISA-based mutational analysis confirmed the intermolecular interaction at antirepressive site 1 observed in the GmaR–MogR structure (Figure 4C). Individual mutation of the MogR N154, I155 and F156 residues to alanine reduced the GmaR_FL_-binding activity of MogR_D_ by 5- to 6-fold. The MogR K152 and T153 residues at site 1 also contributed to GmaR binding but at lower levels than the MogR N154, I155 and F156 residues.

In contrast to site 1, which is located in the middle of the GmaR antirepressor domain, antirepressive site 2 is positioned in the C-terminal region of the antirepressor domain. In the GmaR–MogR structure, GmaR residues from the α26 and α27 helices and their connecting loop make contacts with MogR main body residues at the H6 and H7 helices (Figure 4A and D). However, the individual alanine mutation of site-2 residues did not significantly reduce the GmaR_FL_-binding activity of MogR_D_ in the ELISA. Moreover, the MogR_D_^S114A/Q117A/N118A/K122A^ mutant, in which four site-2 residues (S114, Q117, N118 and K122) were simultaneously changed to alanine, displayed only a 2-fold lower GmaR_FL_-binding activity in the ELISA compared to that of MogR_D_ (Figure 4E). Notably, this alanine mutation analysis addresses the contribution of side chains, but not of main chains, to binding. However, it is obvious that site 2 makes a substantially lower contribution to binding than site 1 despite the larger buried surface area of site 2 in the GmaR–MogR structure.

### Comparative analysis of GmaR–MogR binding and MogR–dsDNA binding

**Figure 5.**
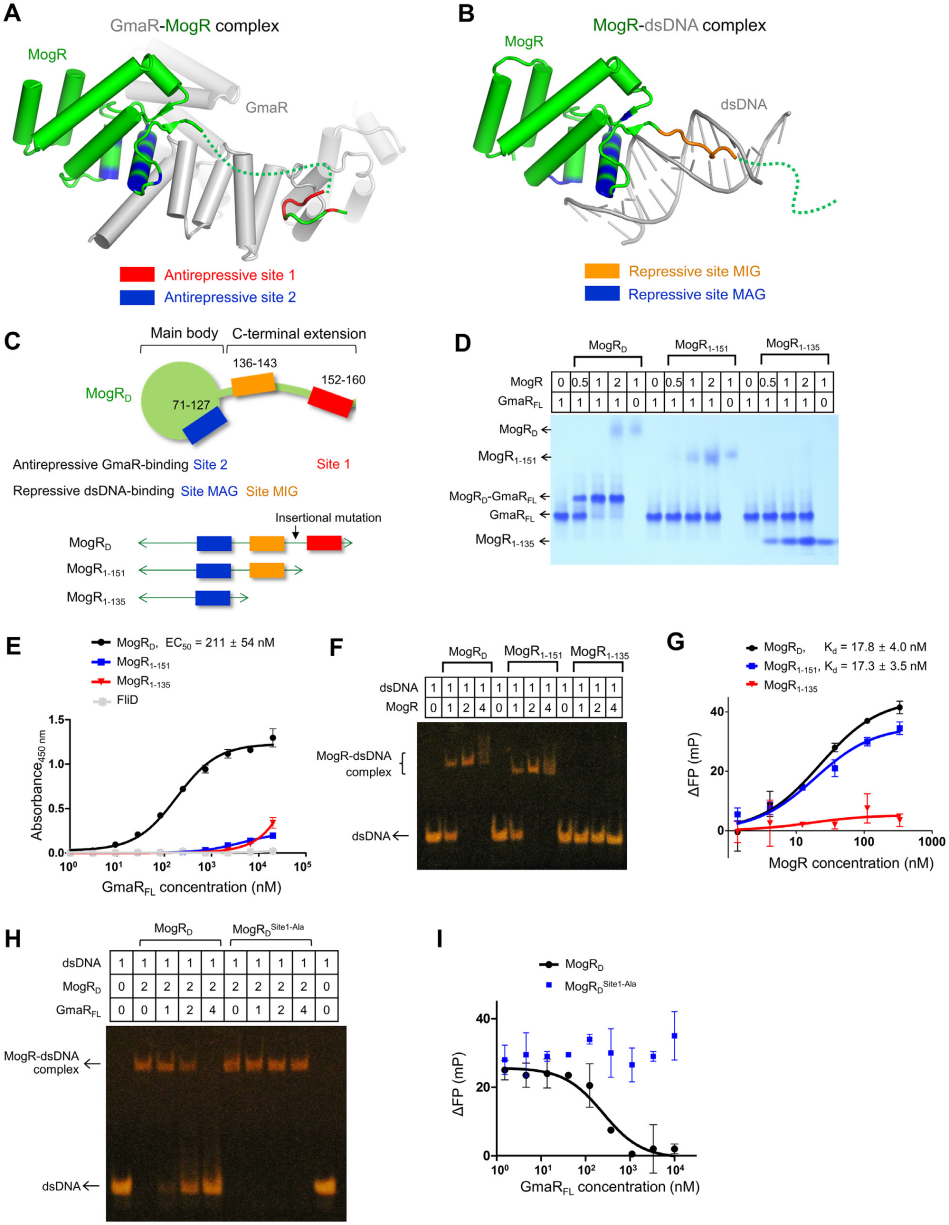
Primary contributions of spatially distinct antirepressive site 1 and repressive site MIG to the GmaR–MogR and MogR–dsDNA interactions, respectively. (**A**) Antirepressive GmaR-binding sites of MogR in the GmaR–MogR complex structure. MogR residues at antirepressive sites 1 and 2 are colored red and blue, respectively, on MogR (green cartoon) in complex with GmaR (gray cartoon). (**B**) Repressive dsDNA-binding sites of MogR in the MogR–dsDNA complex structure (PDB ID: 3FDQ). MogR residues at repressive sites MIG and MAG are colored orange and blue, respectively, on MogR (green cartoon) in complex with dsDNA (gray cartoon). (**C**) Schematic diagram of antirepressive and repressive sites in MogR_D_. Antirepressive site 2 overlaps with repressive site MAG in the main body of MogR. However, antirepressive site 1 and repressive site MIG are segregated in the C-terminally extended coil structure of MogR. Three C-terminally truncated MogR proteins (MogR_D_, MogR_1-151_ and MogR_1-135_) were used in binding assays to define the contribution of each site to GmaR or dsDNA binding. (**D,E**) Native PAGE (D) and ELISA (E) of GmaR_FL_ in the presence of the MogR_D_, MogR_1-151_ or MogR_1-135_ variants to assess the contributions of antirepressive sites 1 and 2 to GmaR–MogR binding (*n* = 3 independent experiments). (**F,G**) EMSA (F) and FP assay (G) of the operator dsDNA in the presence of the MogR_D_, MogR_1-151_ or MogR_1-135_ variants to define the contributions of repressive sites MIG and MAG to MogR–dsDNA binding (*n* = 4 independent experiments). (**H,I**) Critical role of antirepressive site 1 in the inhibitory activity of GmaR on the MogR–dsDNA interaction. The interaction of MogR_D_ or MogR_D_^Site1-Ala^ with dsDNA was analyzed by EMSA (H; *n* = 3 independent experiments) and FP assay (I; *n* = 9 independent experiments) in the absence or presence of GmaR_FL_.

A comparative analysis of the GmaR–MogR structure and the previously reported MogR–dsDNA complex structure (PDB ID: 3FDQ) indicates that MogR uses a common region in the main body and also employs different regions in the C-terminal extension to interact with its binding partners, GmaR and dsDNA (Figure 5A–C) (16). In the MogR–dsDNA structure, MogR makes contacts with the operator dsDNA using two separated regions, repressive sites MAG and MIG (16). Repressive site MAG is located at the H6-H7 region of the MogR main body and interacts with the major groove of dsDNA. Noticeably, in overlaid MogR^dsDNA^ and MogR^GmaR^ structures, repressive site MAG mostly overlaps with antirepressive site 2. In the MogR–dsDNA structure, MogR also recognizes the minor groove of dsDNA using repressive site MIG, corresponding to MogR residues 136–143, in the C-terminal extension. Repressive site MIG is separated from antirepressive sites 1 and 2 in both the primary and tertiary structures of MogR although repressive site MAG is colocalized with antirepressive site 2.

To reveal how MogR engages each intermolecular site during complex formation, C-terminally truncated MogR proteins (MogR_D_, MogR_1-151_ and MogR_1-135_) with different intermolecular binding sites were generated and assessed for GmaR_FL_ binding via native PAGE and ELISA and for dsDNA binding via EMSA and fluorescence polarization (FP) assay (Figure 5D–G). Among the MogR variants, MogR_D_, which retains both antirepressive sites 1 and 2, exhibited the most potent binding to GmaR_FL_ (Figure 5D and E). In contrast, the MogR_1-135_ and MogR_1-151_ variants contain only one antirepressive site, site 2 and displayed negligible binding to GmaR_FL_ at concentrations below 1 μM, indicating that antirepressive site 1 is indispensable for GmaR–MogR complexation. In the MogR–dsDNA binding assays, the MogR_D_ and MogR_1-151_ variants that contain both repressive sites MAG and MIG similarly interacted with dsDNA with a high affinity, whereas the MogR_1-135_ variant that harbors only repressive site MAG without site MIG displayed substantially weaker binding to dsDNA, underscoring an essential role of repressive site MIG in dsDNA recognition by MogR (Figure 5F and G).

The critical, selective roles of sites 1 and MIG in complex formation were further verified by introducing multiple alanine mutations into MogR_D_ at site 1 or site MIG. The MogR_D_^Site1-Ala^ mutant, in which five consecutive MogR residues (residues 152–156) at antirepressive site 1 were replaced with alanine, displayed substantially lower GmaR_FL_-binding affinity than MogR_D_ in native PAGE and ELISA analyses without a significant change in dsDNA binding (Supplementary Figure S9). The essential role of site 1 in GmaR–MogR binding was also verified in cell-based assays, including a pull-down assay and a two-hybrid interaction assay. In the Ni-NTA pull-down assay in *E. coli* cells, the His_6_-tagged GmaR_FL_ protein was obtained in complex with MogR_D_ but not with MogR_D_^Site1-Ala^ or MogR_1-151_ (Supplementary Figure S10). Moreover, in the *E. coli* two-hybrid interaction assay, a high β-galactosidase activity that corresponds to the strong GmaR–MogR interaction was observed for the GmaR_FL_–MogR_D_ pair at 30°C (Supplementary Figure S11). However, when site 1 residues were mutated to alanine (MogR_D_^Site1-Ala^) or removed (MogR_1-151_), the β-galactosidase activity was negligible at 30°C. These observations consistently indicate that site 1 is indispensable for the GmaR–MogR interaction. Furthermore, the inhibitory effect of GmaR_FL_ on the MogR_D_–dsDNA interaction was not observed with the MogR_D_^Site1-Ala^ mutant in the EMSA and FP assay although MogR_D_^Site1-Ala^ contains an intact antirepressive site 2, demonstrating that the site 1 interaction is required for the antirepressive function of GmaR (Figure 5H and I). To confirm the essential role of repressive site MIG in the MogR–dsDNA interaction, the MogR_D_^SiteMIG-Ala^ mutant was generated by mutating five consecutive site-MIG residues (residues 140–144) to alanine. As expected, the MogR_D_^SiteMIG-Ala^ mutant lost dsDNA-binding capacity but displayed a similar GmaR_FL_-binding affinity to that of MogR_D_ (Supplementary Figure S9). Overall, we conclude that spatially distinct sites 1 and MIG in the C-terminal extension of MogR_D_ make primary contributions to binding, compared to the overlapping sites 2 and MAG in the main body of MogR_D_.

### Molecular mechanism of GmaR-mediated inhibition of MogR–dsDNA binding

**Figure 6.**
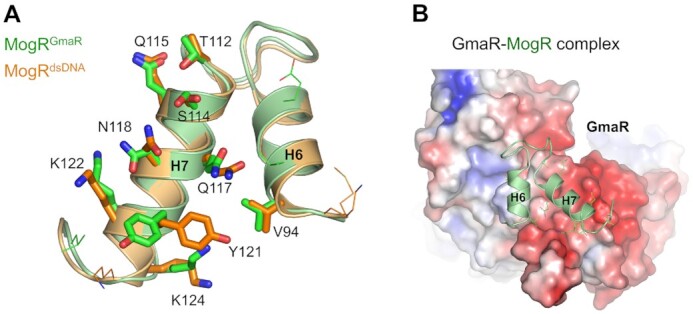
Direct competition mechanism used by antirepressive site 2 of GmaR to interfere with the MogR–dsDNA interaction. (**A**) MogR residues shared by antirepressive site 2 and repressive site MAG. The GmaR-binding residues of MogR at site 2, in particular from the H6 and H7 helices, are also used to interact with dsDNA as site MAG, indicating that GmaR directly competes with dsDNA to interact with site 2 of MogR. In the overlaid MogR^GmaR^ (light green ribbons) and MogR^dsDNA^ (light orange ribbons) structures, the MogR residues that are commonly located at sites 2 (green) and MAG (orange) are represented by sticks, and those observed at only one site are depicted as lines. (**B**) Negative electrostatic potentials of GmaR around the H7 helix of MogR at antirepressive site 2 in the GmaR–MogR complex structure. The electrostatic potentials of GmaR are shown as differently colored surfaces (negative, red surface; neutral, white surface; positive, blue surface). The H6 and H7 helices and their connecting loop of MogR are shown as light green ribbons.

A further comparative structural analysis combined with a structure-based mutational study allows us to propose the regulatory mechanism by which GmaR modulates the MogR–dsDNA interaction and mediates its antirepressive function. In the overlaid MogR^GmaR^ and MogR^dsDNA^ structures, nine MogR residues among the 12 site-2 residues also participate in dsDNA binding at the MAG site (Figure 6A). Thus, these overlapping residues at sites 2 and MAG contribute to selecting between the binding partners, GmaR or dsDNA, through a direct competition mechanism. Consistently, the site-2 interaction between GmaR_FL_ and MogR_1-135_ was inhibited by the operator dsDNA in ELISA, suggesting that GmaR and dsDNA compete with each other to bind MogR at site 2 or site MAG (Supplementary Figure S12). Notably, the common residues of MogR at sites 2 and MAG are mainly found in the H7 helix. The H7 helix is inserted into the major groove of negatively charged dsDNA as a recognition helix in the MogR–dsDNA structure (Figure 5B). In the GmaR–MogR structure, the H7 helix is accommodated by the interhelical space of GmaR between the α26 and α27 helices, which exhibits negative electrostatic potentials (Figures 5A and 6B). These observations suggest that the MogR H7 helix interacts with GmaR and dsDNA via shape and electrostatic complementarities.

**Figure 7.**
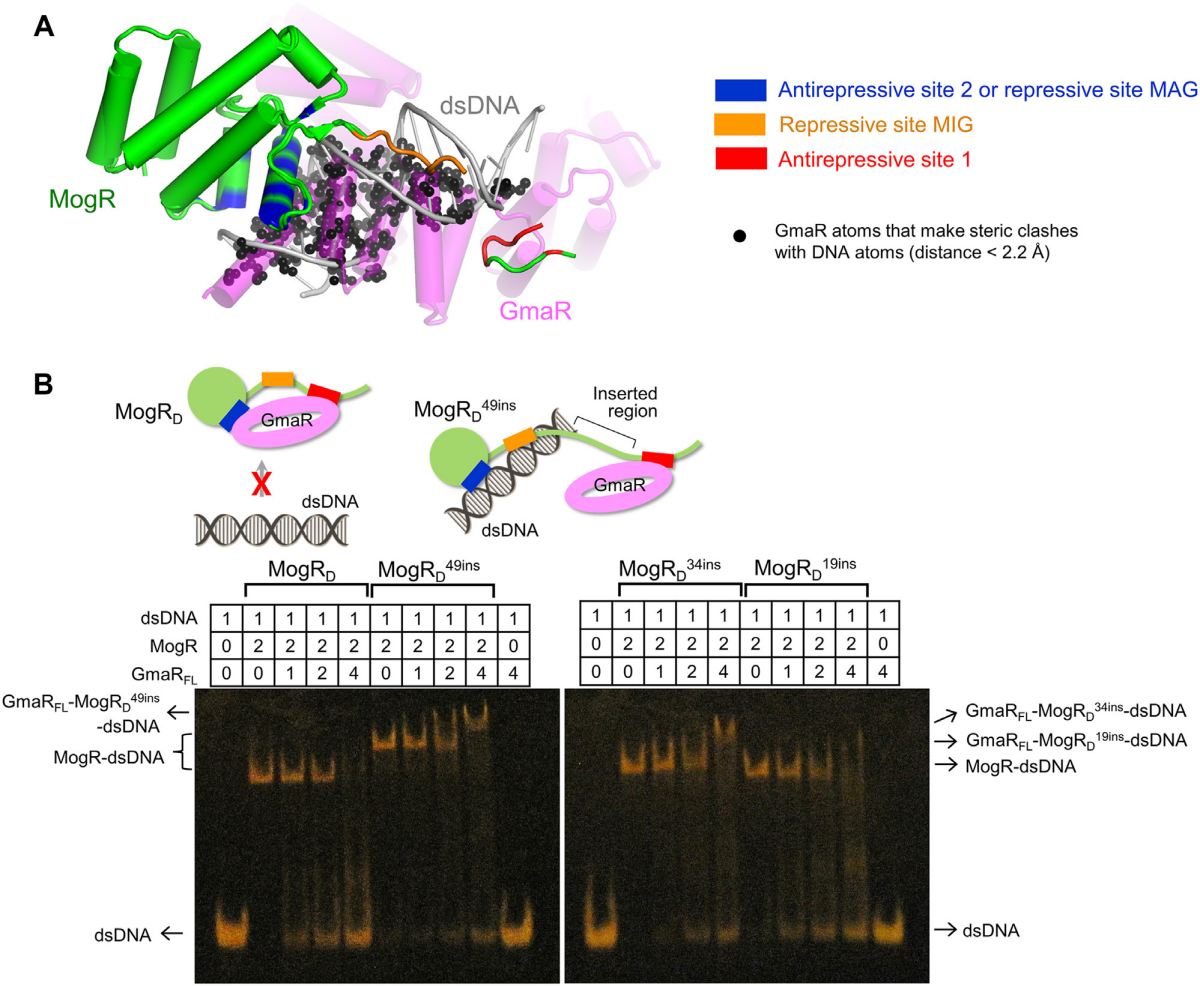
Spatial restraint mechanism used by antirepressive site 1 of GmaR to inhibit the MogR–dsDNA interaction. (**A**) Occlusion of MogR site MIG from dsDNA by the site 1 interaction. The GmaR–MogR and MogR–dsDNA structures are overlaid by superimposing the MogR structures as a reference point. Sites 2/MAG, site MIG and site 1 are colored blue, orange, and red, respectively, in the MogR structures (green) in complex with GmaR (magenta) or dsDNA (gray). In the overlaid structures, GmaR atoms between sites 1 and 2 make substantial steric clashes (black spheres) with dsDNA atoms, particularly at site MIG, suggesting that the site 1 GmaR–MogR interaction occludes MogR site MIG from binding dsDNA despite the distinct positions of sites 1 and MIG and inhibits the minor groove interaction through a spatial restraint mechanism. (**B**) Verification of the spatial restraint mechanism. To alleviate the spatial restraint caused by the site 1 interaction, 49, 34 or 19 additional residues were inserted between sites MIG and 1 in MogR_D_ to generate the MogR_D_^49ins^, MogR_D_^34ins^ and MogR_D_^19ins^ mutants, respectively. The interaction of MogR_D_ or its insertional mutants with dsDNA was analyzed by EMSA in the absence or presence of GmaR_FL_ (*n* = 3 independent experiments). The insertional mutations attenuated the inhibitory effect of GmaR_FL_ on the MogR_D_–dsDNA interaction. Complex formation by MogR_D_ (left) or MogR_D_^49ins^ (right) is schematically shown above the gel image. When MogR_D_ forms a heterodimer with GmaR, MogR_D_ does not interact with dsDNA due to the spatial restraint induced by the site 1 interaction. However, the MogR_D_^49ins^ mutant simultaneously binds GmaR and dsDNA because of relieved spatial restraint.

In contrast to overlapping sites 2 and MAG, MogR sites 1 and MIG, which play key roles in GmaR and dsDNA recognition, respectively, are distantly located. Therefore, GmaR site 1 appears to use a unique antirepression mechanism that differs from the direct competition mechanism of GmaR site 2. In the overlaid GmaR–MogR and MogR–dsDNA structures, site 1-adjacent GmaR residues substantially clash with dsDNA atoms at the MIG site, suggesting that the site 1 interaction limits the accessibility of dsDNA to the MIG site of MogR via a spatial restraint (Figure 7A). To address whether the site 1 interaction uses a spatial restraint to abrogate the MogR–dsDNA interaction, MogR_D_ was engineered to alleviate the spatial restraint by inserting 19, 34 and 49 additional residues (MogR_D_^19ins^, MogR_D_^34ins^ and MogR_D_^49ins^ mutants, respectively) between sites MIG and 1 (Figures 1A and 5C). These insertional mutants all displayed dsDNA- and GmaR_FL_-binding affinities comparable to those of MogR_D_ in the FP assay and ELISA, respectively (Supplementary Figure S13). However, the insertional mutations mitigated the inhibitory activity of GmaR_FL_ on MogR–dsDNA binding in proportion to the number of inserted residues in the EMSA (Figure 7B). As a result of the alleviation of spatial restraint, the insertional mutants of MogR_D_ formed a heterotrimeric complex with GmaR_FL_ and dsDNA. Based on structural and mutational analyses, we propose that GmaR combines direct competition and spatial restraint mechanisms to completely inhibit the interaction of MogR with dsDNA.

### Temperature-dependent changes in the oligomeric state and function of GmaR

**Figure 8.**
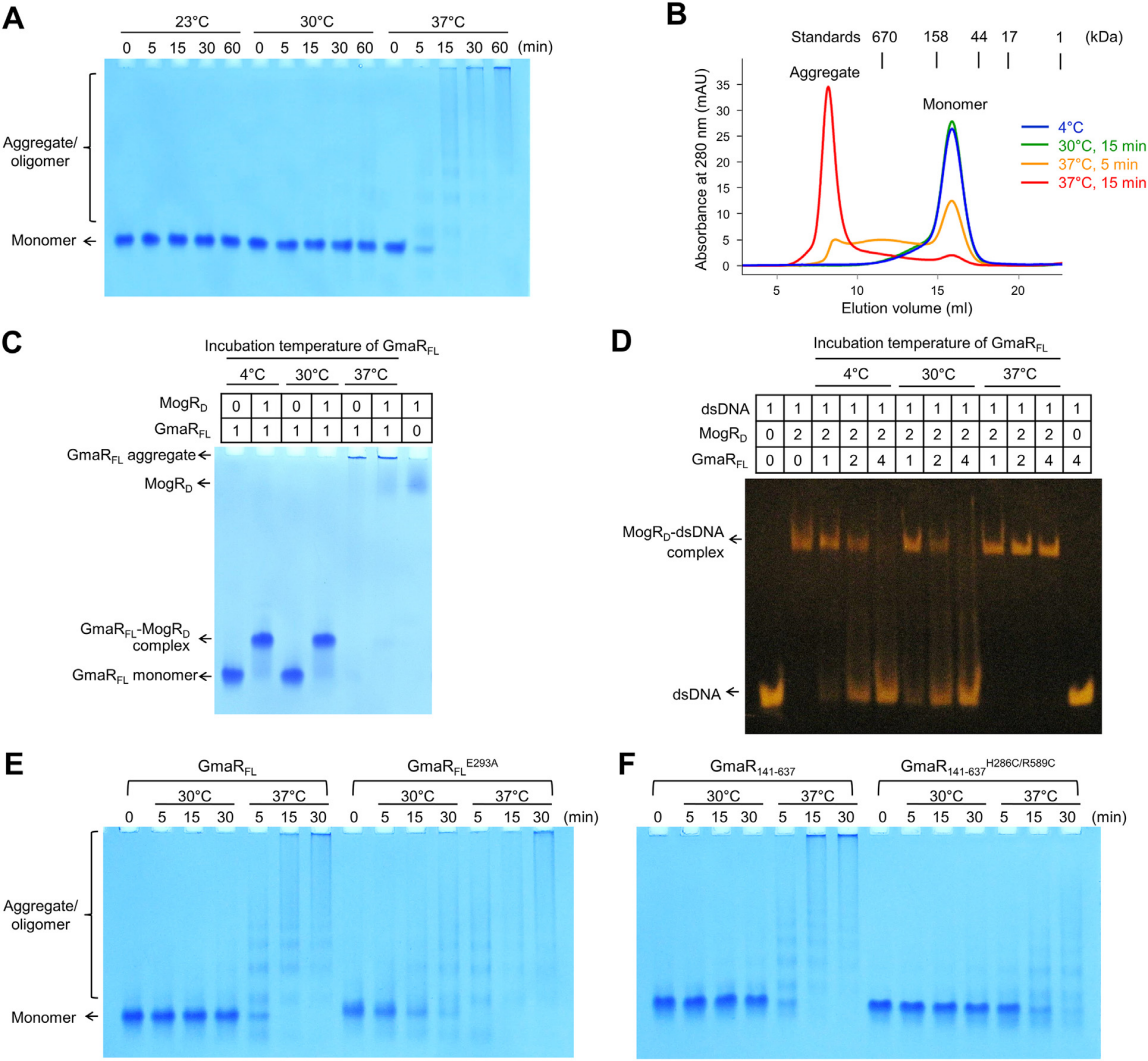
The 37°C-labile stability and function of GmaR. (**A**) 37°C-induced aggregation of GmaR_FL_ in native PAGE (*n* = 3 independent experiments). The monomeric GmaR_FL_ protein was incubated at 23, 30 or 37°C for various times and then analyzed by native PAGE to address a temperature-dependent change in the oligomeric state of GmaR_FL_. (**B**) 37°C-induced aggregation of GmaR_FL_ in gel-filtration chromatography (*n* = 3 independent experiments). The monomeric GmaR_FL_ protein was incubated at 30 or 37°C for 5 or 15 min and then analyzed by gel-filtration chromatography to address a temperature-dependent change in the oligomeric state of GmaR_FL_. (**C**) MogR-binding activity of GmaR at different temperatures. The GmaR_FL_ protein was incubated at 4, 30 or 37°C for 15 min, and then the interaction of the resulting GmaR_FL_ protein with MogR_D_ was analyzed by native PAGE (*n* = 4 independent experiments). (**D**) Inhibitory effect of GmaR on the MogR–dsDNA interaction depending on exposure temperature. The GmaR_FL_ protein was incubated at 4, 30 or 37°C for 15 min, and then the inhibition of the MogR–dsDNA interaction by the resulting GmaR_FL_ protein was analyzed by EMSA (*n* = 4 independent experiments). (**E**) Aggravated aggregation of GmaR at 30 and 37°C resulting from a loss of interdomain contacts at the GmaR E293 residue. The GmaR_FL_ and GmaR_FL_^E293A^ proteins were incubated at 30 or 37°C, and their oligomeric states were analyzed by native PAGE (*n* = 3 independent experiments). (**F**) Alleviated aggregation of GmaR at 37°C by stabilizing the interdomain interaction between the GmaR H286 and R589 residues. The GmaR_141-637_ and GmaR_141-637_^H286C/R589C^ proteins were incubated at 30 or 37°C, and their aggregation profiles were determined by native PAGE (*n* = 3 independent experiments). GmaR_141-637_ was used for the H286C and R589C mutations instead of GmaR_FL_ to avoid unexpected disulfide bond formation caused by three cysteine residues (residues 8, 87 and 139) in the glycosyltransferase domain. GmaR_141-637_ exhibited thermostability similar to that of GmaR_FL_.

The antirepressive function of GmaR on MogR-mediated transcriptional control has been shown to depend on the growth temperature of *L*. *monocytogenes* (14,17,18). A temperature change dynamically affects the levels of the functional GmaR protein via transcriptional and posttranslational regulation. To address the temperature-sensitive posttranslational modulation of the GmaR protein, we assessed the protein stability of GmaR by native PAGE and gel-filtration chromatography at 23, 30 and 37°C. The GmaR_FL_ protein exhibited a stark contrast in the oligomeric state depending on the temperature (Figure 8A and B). The GmaR_FL_ protein existed as a monomer at 23 and 30°C. However, upon incubation at 37°C, the molecular size of GmaR_FL_ increased as high-order oligomers and aggregates. The 37°C-induced aggregation of GmaR was correlated with a loss of its antirepressive function. The GmaR_FL_ protein pretreated at 37°C aggregated and lost its ability to bind MogR and interfere with the MogR–dsDNA interaction (Figure 8C and D; Supplementary Figure S14). In contrast, the GmaR_FL_ protein preincubated at 30°C remained monomeric and retained its MogR-binding functions (Figure 8C and D). This temperature-based analysis indicates that GmaR is a thermosensitive protein that forms nonfunctional aggregates in response to a temperature increase.

GmaR forms a unique circular structure through intramolecular interdomain interactions, as observed in the GmaR structures obtained from crystals grown at 18°C (Figure 2). Notably, the interdomain interaction of GmaR is characterized by relatively small binding interfaces (∼330 Å^2^) and thus expected to be easily destabilized by an environmental change. Based on these observations, we hypothesized that the interdomain interaction is required to maintain the circular conformation of GmaR at low temperatures but is vulnerable to a temperature increase. To assess the contribution of the GmaR interdomain interaction to GmaR thermostability, we disrupted the interdomain interaction by mutation and monitored mutation-induced changes in aggregation at 30 and 37°C (Figure 8E). In the GmaR structure, the E293 residue of GmaR participates in the interdomain interaction through hydrogen bonds and a salt bridge with the R617 residue (Figure 2B). When the E293 residue was replaced with alanine in GmaR_FL_, GmaR aggregate formation was exacerbated at 37°C, and aggregates were observed even at 30°C, presumably due to a defect in the interdomain interaction (Figure 8E). Moreover, when pretreated at 30°C, the GmaR_FL_^E293A^ mutant exhibited a lower MogR_D_-binding level and a less potent inhibitory effect on the MogR_D_–dsDNA interaction in native PAGE and EMSA analyses, respectively, than observed with the GmaR_FL_ protein (Supplementary Figure S15A and B). Consistently, in the *E. coli* two-hybrid interaction assay, a lower β-galactosidase activity indicating a lower GmaR–MogR binding level was observed for the GmaR_FL_^E293A^ mutant at 30°C than for GmaR_FL_ presumably because the level of the GmaR monomer protein that was available for MogR binding decreased through the E293A mutation-mediated destabilization of GmaR protein (Supplementary Figure S11). Conversely, the interdomain interaction of GmaR was artificially stabilized by introducing an interdomain disulfide bond. To generate the interdomain disulfide bond, the adjacent H286 and R589 residues from the interdomain interfaces of the GmaR TPR and antirepressor domains, respectively, were replaced with cysteine residues in GmaR_141-637_ (Figure 2B). GmaR_141-637_^H286C/R589C^ mutant aggregation was reduced at 37°C, and the mutant displayed enhanced MogR_D_-binding capacity and more potent inhibitory activity against the MogR_D_–dsDNA interaction than that observed with the GmaR_141-637_ protein (Figure 8F; Supplementary Figure S15C and D). Collectively, these findings indicate that the interdomain interaction is a critical determinant that regulates the protein stability and the antirepressive function of GmaR in a temperature-dependent manner.

## DISCUSSION

**Figure 9.**
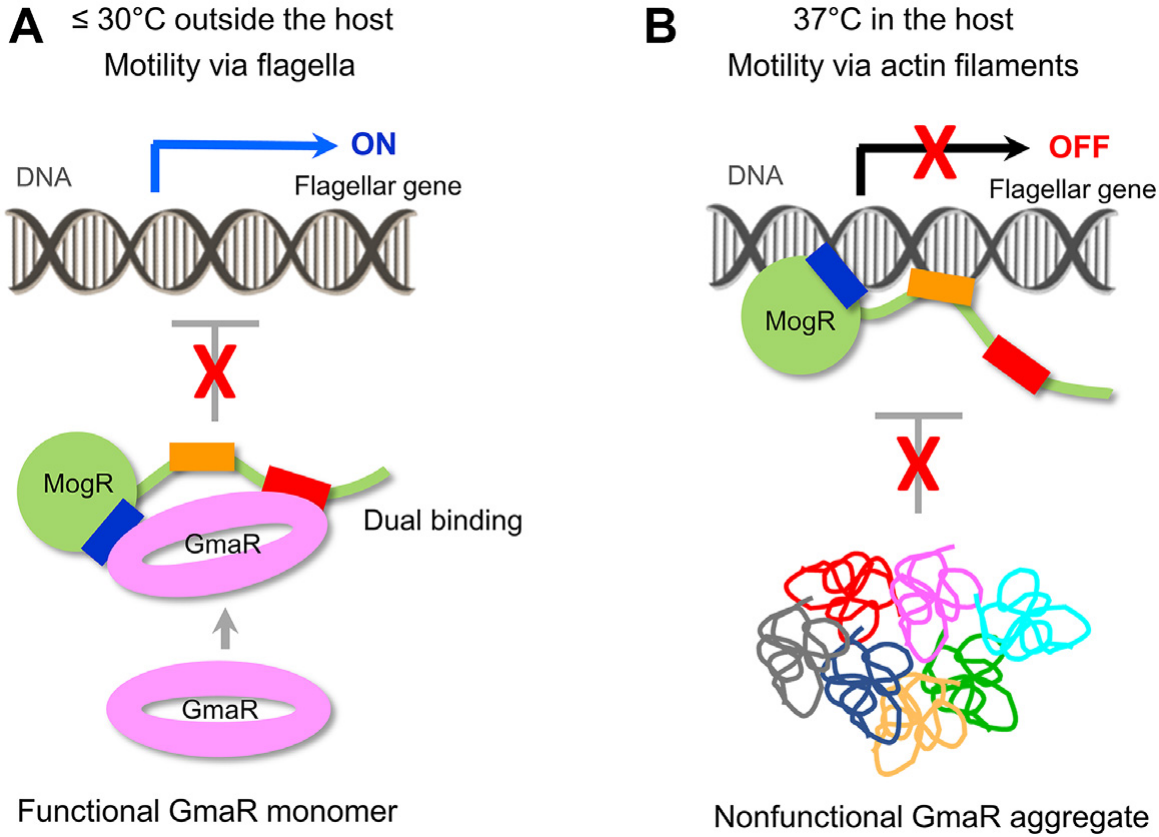
Proposed molecular model for the temperature-dependent regulation of flagellar expression by the GmaR–MogR system. (**A**) Flagellar expression through GmaR-mediated neutralization of MogR at or below 30°C outside the host. (**B**) GmaR aggregation and MogR-mediated suppression of flagellar expression at 37°C inside the host.

Extensive structural and biochemical analyses of GmaR and MogR allowed us to propose a molecular model for the temperature-dependent regulation of flagellar expression through the GmaR–MogR system (Figure 9). At or below 30°C, GmaR exists as a functional monomer in a circularly enclosed conformation. The monomeric GmaR protein interacts with MogR through a dual binding mode and counteracts the inhibitory activity of MogR on flagellar expression. As a result of GmaR-mediated derepression of flagellar expression, *L. monocytogenes* generates flagella and moves through flagellum-based locomotion. However, at 37°C, monomeric GmaR is modified into a nonfunctional aggregate. Upon aggregation, GmaR is not able to recognize and neutralize MogR. Thus, MogR interacts with operator DNA and transcriptionally represses flagellar expression. Consequently, *L. monocytogenes* does not produce flagella and instead utilizes actin-based motility inside the infected host cell.

The GmaR protein forms aggregates at 37°C. Protein aggregates can be removed through the cellular processes of disaggregation and proteolysis, indicating the possible fate of cellular GmaR protein at 37°C (31). Inside the infected human host, the GmaR protein is expected to aggregate and subsequently undergo degradation. This proposed mechanism is consistent with a previous finding that the GmaR protein expressed at room temperature has a lower survival rate in the cell at 37°C than at 30°C (18). Protein aggregation is generally accompanied by a change in the secondary structure. Indeed, in a previous circular dichroism study, when the temperature shifted from 20 to 38°C, the α-helix content of the GmaR protein decreased from 55% to 40%, and the random coil content instead increased from 38% to 51%, suggesting that α-helices are converted into random coils due to thermal stress (18). Therefore, upon exposure to 37°C, GmaR protein appears to be modified into a partially unfolded conformation, exposing larger hydrophobic surfaces to solvent and promoting intermolecular nonspecific hydrophobic interactions and aggregate formation (32).

GmaR is a four-domain protein that consists of glycosyltransferase, TPR, linker and antirepressor domains. GmaR mediates two different functions as a moonlighting protein primarily using two domains. The N-terminal glycosyltransferase domain of GmaR catalyzes the covalent attachment of the N-acetylglucosamine sugar to the flagellin protein that constitutes the flagellar filament. The C-terminal antirepressor domain of GmaR is required for transcriptional derepression of flagellar genes. What is the biological function of the centrally located TPR domain in GmaR? TPR generally acts as a molecular scaffold that is required for protein-protein interactions (33). The GmaR TPR domain mediates the interdomain interaction with the antirepressor domain as an interaction-promoting scaffold. Such a TPR-mediated interdomain interaction enhances the structural circularity and protein stability of GmaR and facilitates the antirepressor function of GmaR at low temperatures. However, a temperature shift to 37°C destabilizes the TPR domain-mediated interdomain interaction, allowing GmaR to aggregate and lose its antirepressor function. In addition to the intramolecular interdomain interaction, the TPR domain is likely to participate in an intermolecular interaction to specifically recognize the flagellin protein as a substrate of glycosyltransferase, given that O-linked N-acetylglucosamine transferase directly interacts with the intact protein substrate using its N-terminal TPR domain (34). Thus, we propose that the central TPR domain of GmaR is a molecular scaffold that contributes to both glycosyl transfer and temperature-dependent transcriptional derepression.

Antirepressor proteins suppress the activity of a transcriptional repressor using various molecular mechanisms, including oligomerization change, conformational rearrangement, degradation or DNA mimicry (35–41). The GmaR antirepressor neutralizes MogR via a unique dual binding mode. GmaR directly occludes the major groove-binding site of MogR via the site-2 interaction and thus exerts its inhibitory effect on MogR through a direct competition mechanism. Furthermore, GmaR antagonizes the potent interaction of MogR with the minor groove of dsDNA through a spatial restraint mechanism by binding the MogR region near the minor groove interaction site.

In addition to the direct competition and spatial restraint mechanisms, GmaR may modulate the oligomeric state or organization of MogR as a MogR neutralization mechanism, similar to the bacterial SinI and viral Ant antirepressors (37,40,41). Noticeably, in the MogR_D_–dsDNA complex structure, two MogR_D_ monomers simultaneously interact with palindromic dsDNA. The dimeric architecture of MogR on dsDNA cannot be compatible with GmaR because the N-terminal region of GmaR^MogR^ clashes with the second MogR subunit (particularly site MIG) from the superimposed 2:1 MogR–dsDNA complex structure. Therefore, if the dimeric organization of MogR in the MogR_D_–dsDNA structure is maintained even in the absence of dsDNA, GmaR is expected to prevent MogR dimerization, contributing to the inhibition of MogR–dsDNA binding. This structural observation opens a possibility that GmaR may, in addition to the specific interaction mode described in the present study, also modulate the oligomeric state or organization of MogR to mediate its antirepressor function. Interestingly, MogR oligomerizes and simultaneously recognizes multiple DNA sites with assistance of the C-terminal oligomerization domain, suggesting that MogR oligomerization is required for the complete repression of flagellar expression (16). However, to determine whether GmaR directly influences MogR oligomerization as part of its antirepressor function, a further structural study on the oligomeric assembly of MogR_FL_ in the presence or absence of GmaR is required.

## DATA AVAILABILITY

The atomic coordinates and structure factors for GmaR (PDB ID: 7X9R) and its complex with MogR (PDB ID: 7X9S) have been deposited in the Protein Data Bank (www.pdb.org).

## Supplementary Material

gkac815_Supplemental_File
