# Supplementary material for: Structural basis of flagellar motility regulation by the MogR repressor and the GmaR antirepressor in Listeria monocytogenes
Source: Nucleic Acids Res. 2022 Oct 26;50(19):11315–30. doi: 10.1093/nar/gkac815 (PMC9638930; doi:10.1093/nar/gkac815)
Supplement: gkac815_Supplemental_File [file gkac815_supplemental_file.pdf]

Supplementary Figure S1

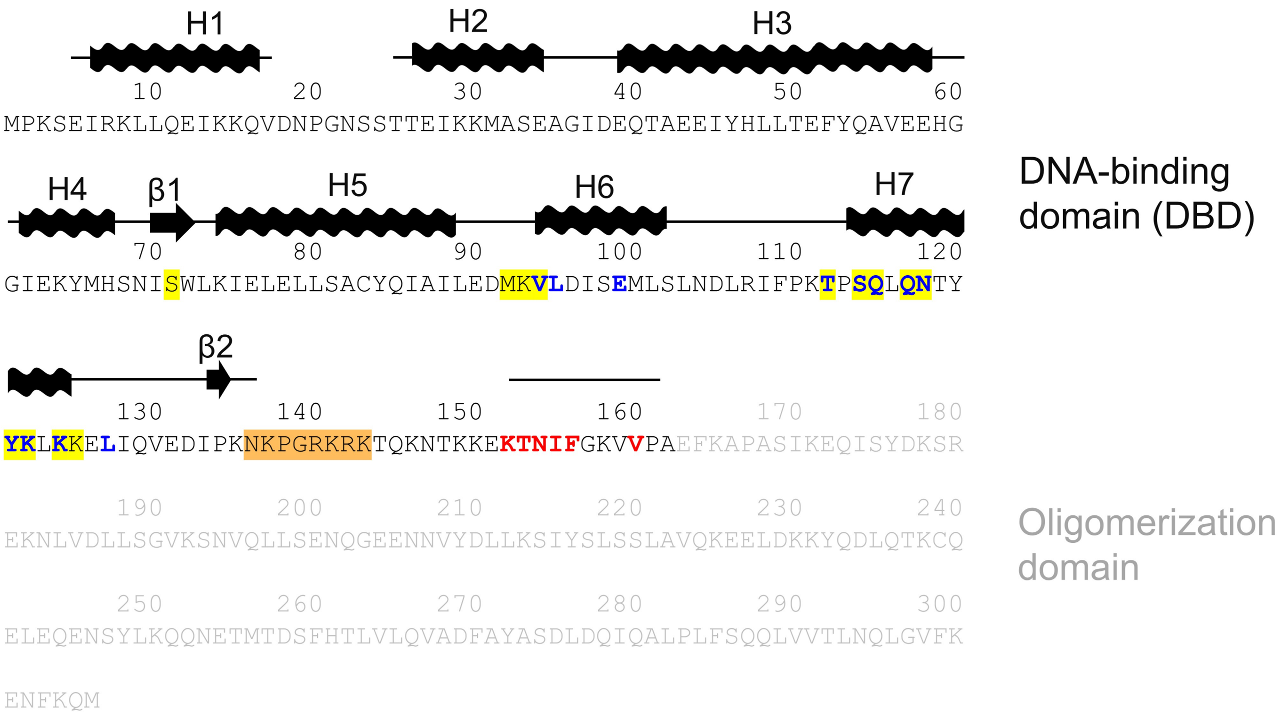

Supplementary Figure S1. Amino acid sequence of MogR. The DBD and oligomerization domain of MogR are colored black and gray, respectively. The MogR residues at antirepressive sites 1 and 2 are shown as red and blue letters, respectively. The MogR residues at repressive sites MAG and MIC are highlighted by yellow and orange backgrounds, respectively. The MogR regions that are built in the GmaR-MogR structure are designated by secondary structural elements (α-helices, waves; β-strands, arrows; coils, lines) above the MogR sequence.

Supplementary Figure S2

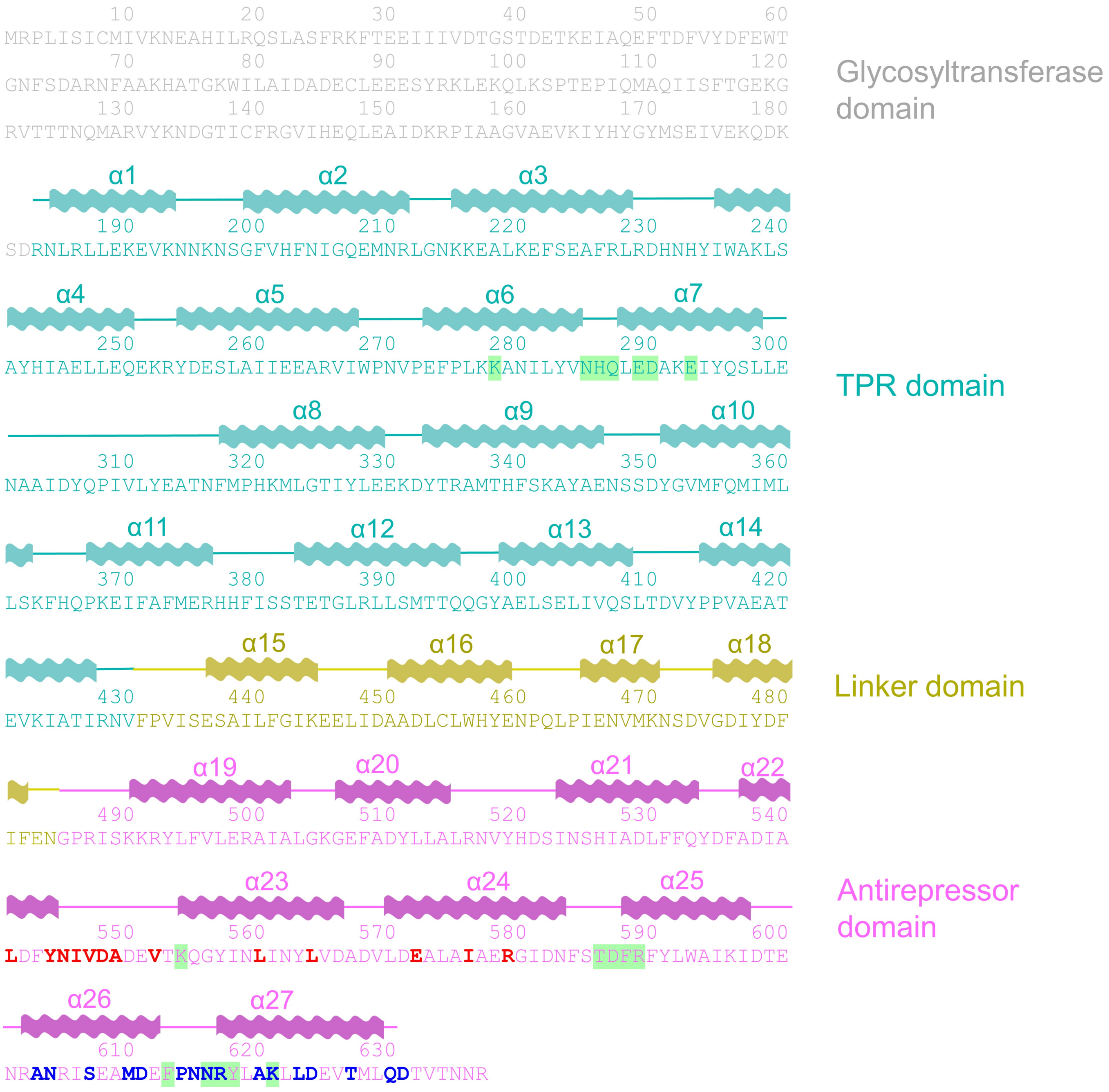

Supplementary Figure S2. Amino acid sequence of GmaR. The glycosyltransferase, TPR, linker, and antirepressor domains are colored gray, cyan, yellow, and magenta, respectively. The GmaR residues at antirepressive sites 1 and 2 are shown as red and blue letters, respectively. The GmaR residues involved in the interdomain interaction are highlighted by green backgrounds. The GmaR regions that are built in the GmaR^{Apo} structure are designated by secondary structural elements (α-helices, waves; coils, lines) above the GmaR sequence.

Supplementary Figure S3

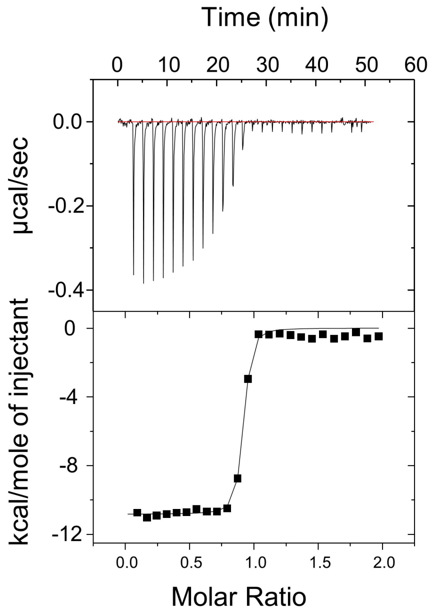

K_d = 21.5 ± 3.2 nM

Supplementary Figure S3. ITC analysis of the interaction between $GmaR_{141\text{-}637}$ and $MogR_D$ (n = 3 independent experiments). $GmaR_{141\text{-}637}$ displayed a similar $MogR_D$-binding affinity (K_d, 21.5 ± 3.2 nM; Wiseman c-value, 1181±188) to that of $GmaR_{FL}$ (K_d, 12.9 ± 4.0 nM) (42).

Supplementary Figure S4

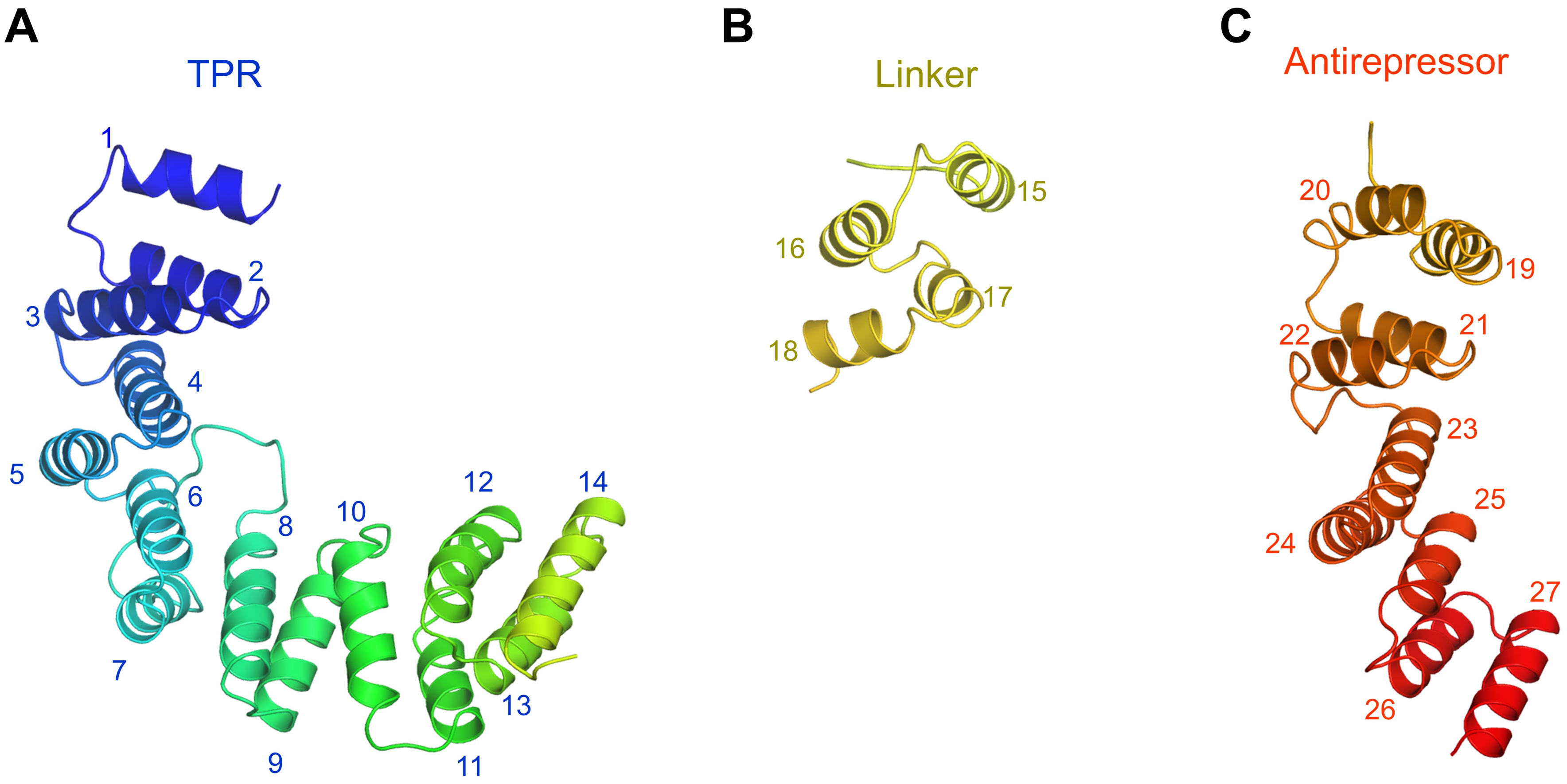

Supplementary Figure S4. Structures of GmaR domains. The structures of the GmaR^{Apo} TPR (A), linker (B), and antirepressor (C) domains are shown as ribbons colored identically to the GmaR^{Apo} structure shown in Figure 2A.

Supplementary Figure S5

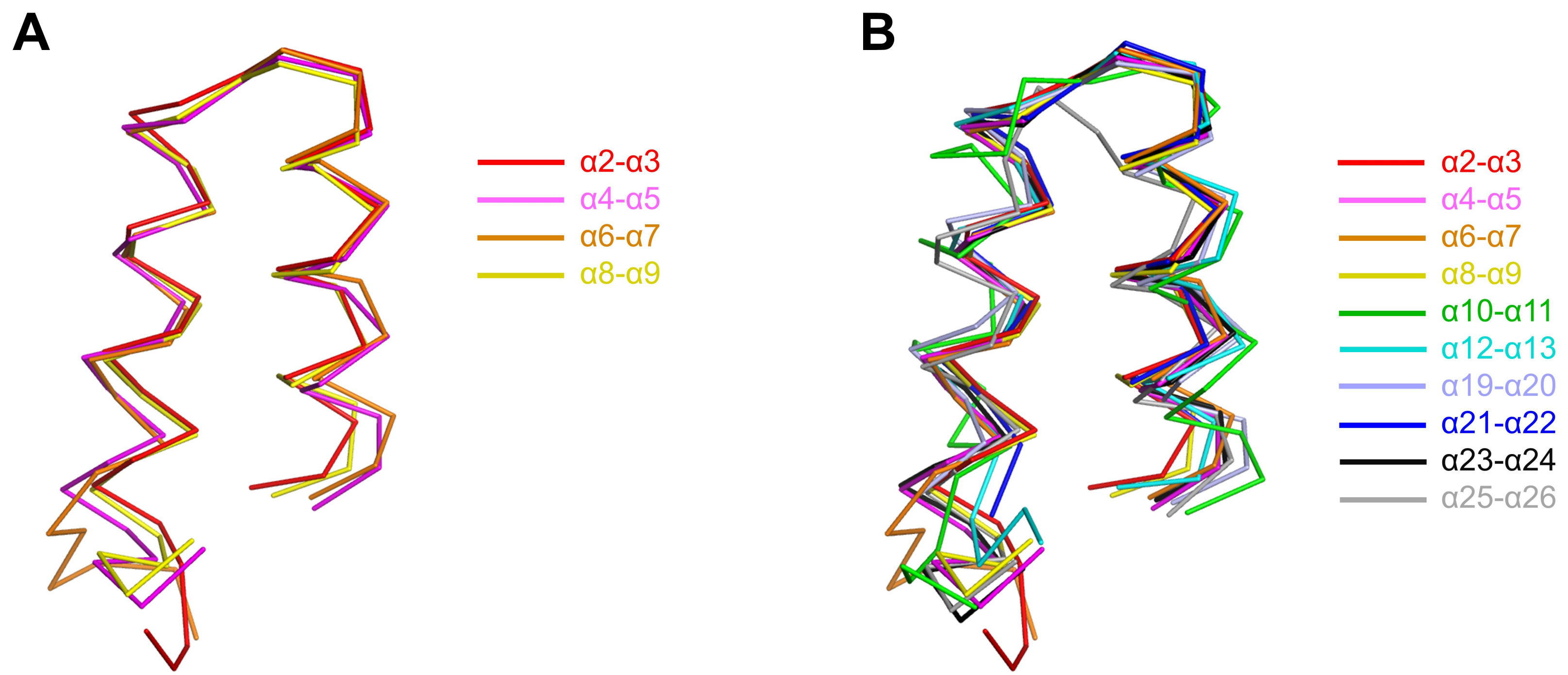

Supplementary Figure S5. Similar structures of HTH repeats present in the TPR and antirepressor domains of GmaR^{Apo}. (**A**) Structural overlays of the HTH repeats from the four TPR motifs in the GmaR^{Apo} TPR domain. (**B**) Structural overlays of HTH repeats from the TPR and antirepressor domains of GmaR^{Apo}.

Supplementary Figure S6

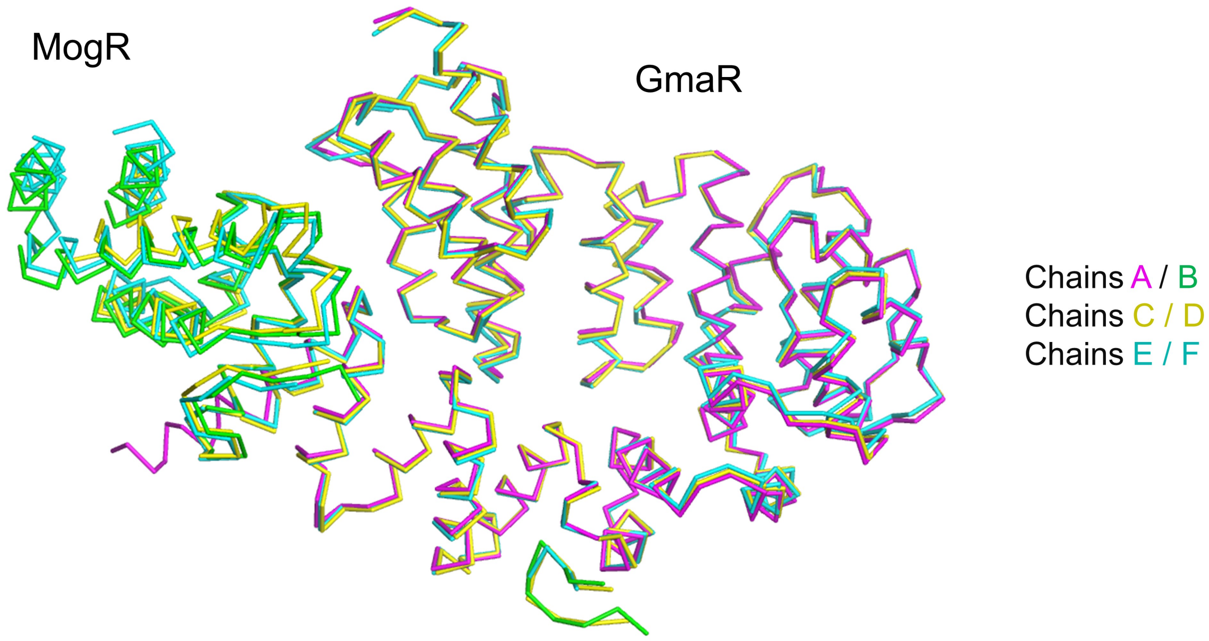

Supplementary Figure S6. Structural overlays of three 1:1 GmaR-MogR complexes in the asymmetric unit of the crystal.

Supplementary Figure S7

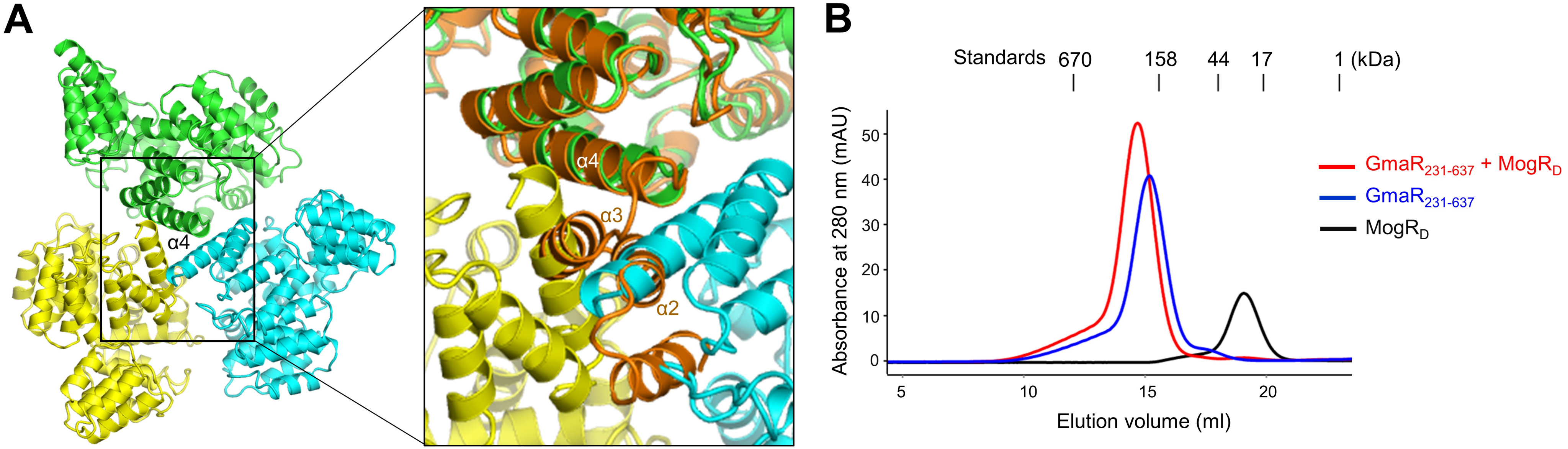

Supplementary Figure S7. Artificial trimeric organization of GmaR$_{231\text{-}637}$. GmaR$_{231\text{-}637}$ forms a trimer (green, cyan, and yellow ribbons) in the crystal structure of the GmaR$_{231\text{-}637}$-MogR$_{D}$ complex (**A**, left panel; MogR$_{D}$ is omitted from the complex structure for clarity) unlike monomeric GmaR$_{FL}$. Consistently, in gel-filtration chromatography, GmaR$_{231\text{-}637}$ (calculated molecular weight, 47.8 kDa) was eluted in a similar volume to that of the 158 kDa standard, and the apparent molecular weight of GmaR$_{231\text{-}637}$ was estimated to be ~160 kDa, indicating that GmaR$_{231\text{-}637}$ forms a trimer in solution (**B**). GmaR$_{231\text{-}637}$ trimerization is primarily mediated by the hydrophobic side of the N-terminal α-helix of GmaR$_{231\text{-}637}$ (α4) (**A**, left panel). However, in the monomeric GmaR$_{141\text{-}637}$ structure (orange ribbons) containing additional N-terminal α helices (α1, α2, and α3), the hydrophobic region of the α4 helix is occluded by the α2 and α3 helices (**A**, right panel). Consistently, when the GmaR$_{231\text{-}637}$ trimer and GmaR$_{141\text{-}637}$ monomer structures are superimposed, the α2 and α3 helices of the GmaR$_{141\text{-}637}$ structure sterically clash with the α4 helices from the other chains of the GmaR$_{231\text{-}637}$ trimer (**A**, right panel). These observations indicate that GmaR$_{231\text{-}637}$ forms a nonbiological trimer to stabilize the hydrophobic side of the α4 helix that is inevitably generated by eliminating the N-terminal region of GmaR (residues 1-230). Although GmaR$_{231\text{-}637}$ forms a trimer unlike GmaR$_{FL}$, GmaR$_{231\text{-}637}$ displayed a MogR$_{D}$-binding ability based on the results of gel-filtration chromatography (**B**). The data of gel-filtration chromatography are representative of three independent experiments that yielded similar results.

Supplementary Figure S8

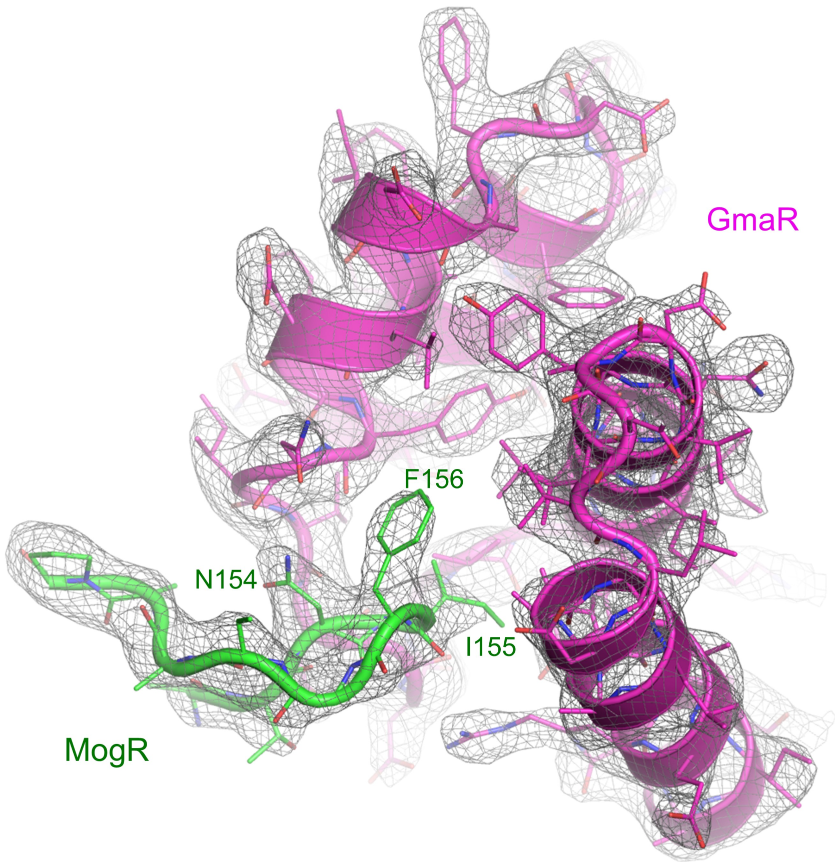

Supplementary Figure S8. Electron density map (gray wires; 1σ in the 2Fo−Fc map) at or near site 1 in the structure of the complex between $GmaR_{231\text{-}637}$ (magenta ribbons and sticks) and $MogR_D$ (green ribbons and sticks). To model the MogR site-1 region in the $GmaR_{231\text{-}637}$-$MogR_D$ complex structure, we searched for an extra electron density corresponding to the missing C-terminal region of $MogR_D$ (residues 137-162) after model building and refinement in GmaR and the main body of $MogR_D$ (residues 5-136). An extra density was identified near the GmaR cavity that is generated by the α22, α23, and α24 helices. Despite the relatively low resolution of the X-ray diffraction data, the extra electron density was undoubtedly visible and sufficient to register MogR residues 152-161. In particular, the three MogR residues (N154, I155, and F156) that play a critical role in GmaR recognition exhibited obvious electron density maps. The only phenylalanine residue (F156) in the C-terminal region of $MogR_D$ was first located in the GmaR cavity, considering the shape of the extra electron density and chemical properties of its surrounding GmaR residues. Subsequently, we built the N154 and I155 residues and then the remaining residues based on the electron density. The inclusion of MogR residues 152-161 in the complex model reduced R_{work} and R_{free} values by 0.54% and 0.77%, respectively.

Supplementary Figure S9

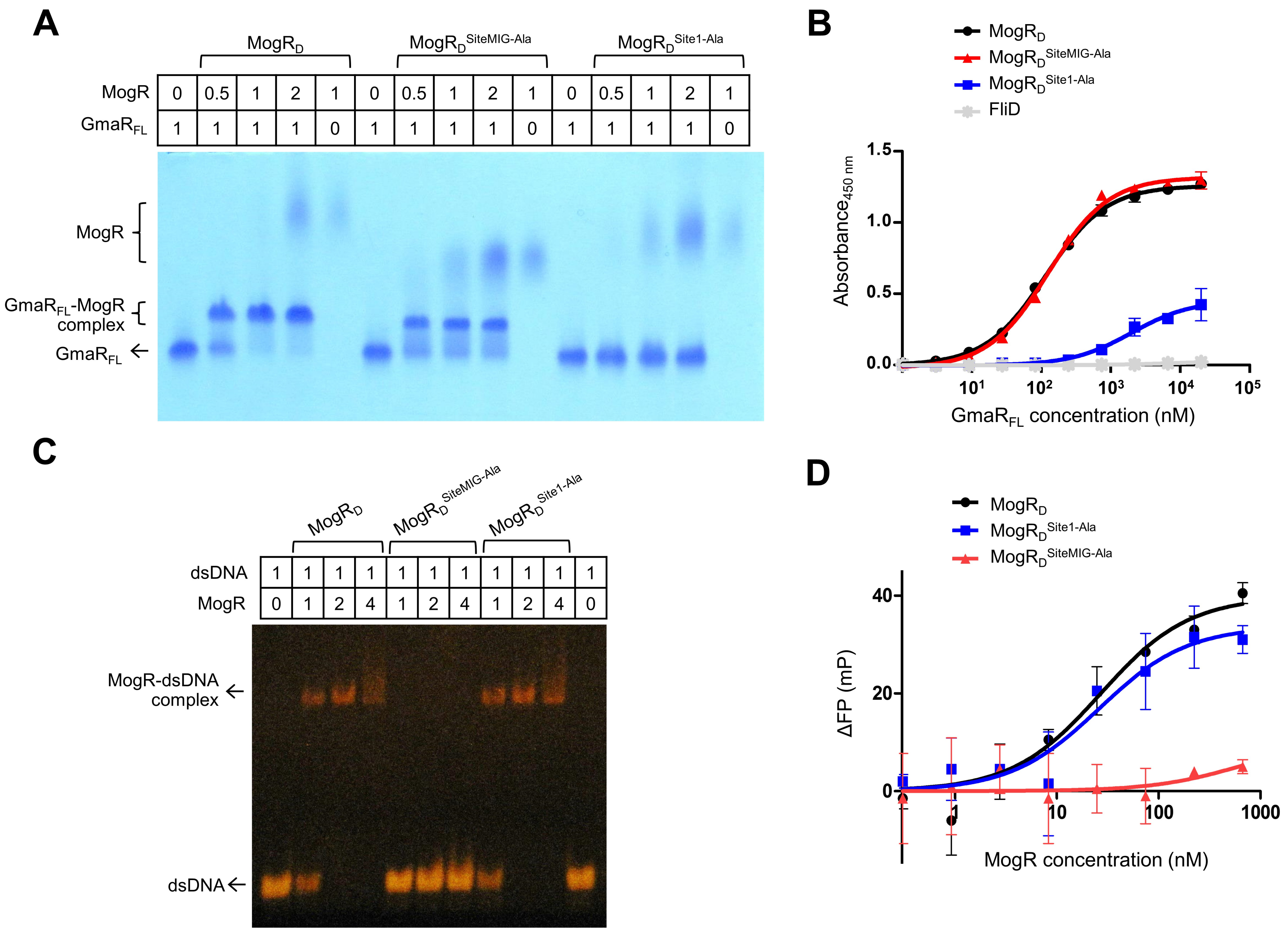

Supplementary Figure S9. Major contributions of MogR site-1 residues 152-156 and MIG site residues 140-144 to the GmaR-MogR and MogR-dsDNA interactions, respectively. (**A, B**) Native PAGE (A) and ELISA (B) analyses of the interaction of $GmaR_{FL}$ with $MogR_D$, $MogR_D^{SiteMIG\text{-}Ala}$, or $MogR_D^{Site1\text{-}Ala}$ (n = 3 independent experiments). (**C, D**) EMSA (C; n = 3 independent experiments) and FP assay (D; n = 4 independent experiments) analyses of the interactions of $MogR_D$, $MogR_D^{SiteMIG\text{-}Ala}$, and $MogR_D^{Site1\text{-}Ala}$ with the operator dsDNA.

Supplementary Figure S10

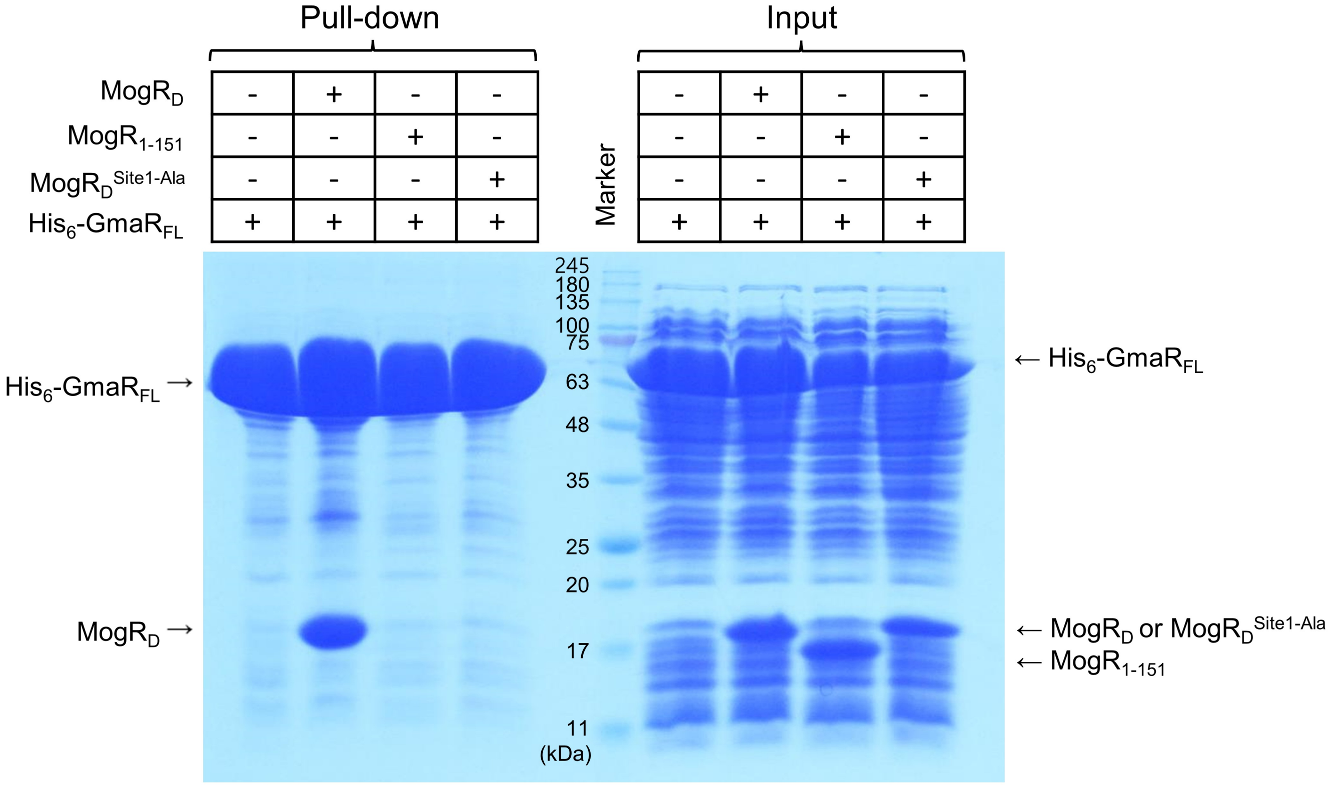

Supplementary Figure S10. Pull-down assay to confirm the critical role of site 1 in the GmaR-MogR interaction (n = 4 independent experiments). The His_6-tagged $GmaR_{FL}$ protein (His_6-$GmaR_{FL}$) was coexpressed with MogR ($MogR_D$, $MogR_{1\text{-}151}$, or $MogR_D^{Site1\text{-}Ala}$) in *E. coli* cells and was purified using Ni-NTA resin. Protein samples obtained before and after Ni-NTA pull-down (input and pull-down, respectively, in the figure) were subjected to SDS-PAGE and Coomassie brilliant blue staining. The His_6-tagged $GmaR_{FL}$ protein was copurified with $MogR_D$ but not with $MogR_{1\text{-}151}$ or $MogR_D^{Site1\text{-}Ala}$, indicating that site 1 is required for the GmaR-MogR interaction.

Supplementary Figure S11

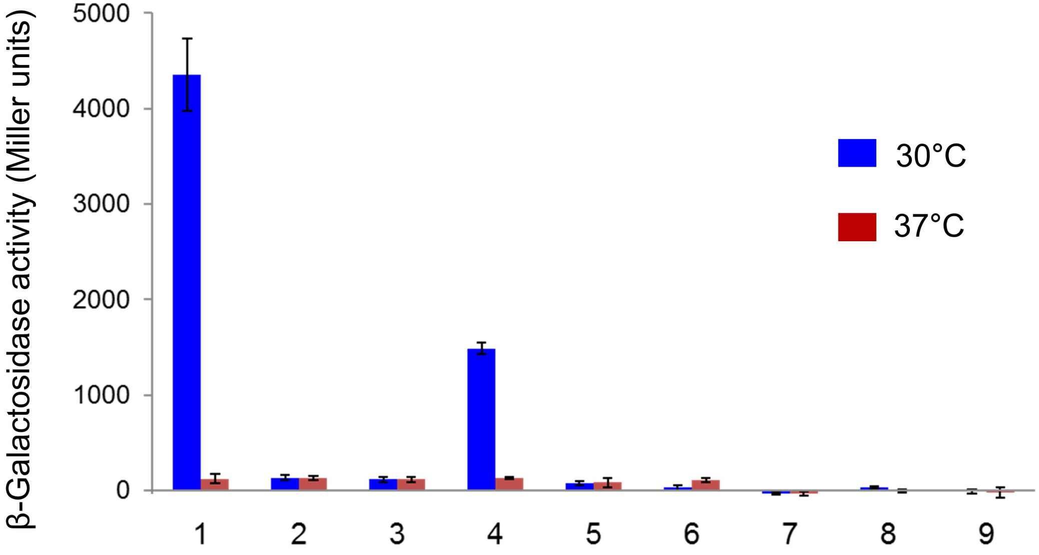

Column	GmaR	MogR
1	$GmaR_{FL}$	$MogR_{D}$
2	$GmaR_{FL}$	$MogR_{D}^{Site1\text{-}Ala}$
3	$GmaR_{FL}$	$MogR_{1\text{-}151}$
4	$GmaR_{FL}^{E293A}$	$MogR_{D}$
5	$GmaR_{FL}$	-
6	$GmaR_{FL}^{E293A}$	-
7	-	$MogR_{D}$
8	-	$MogR_{D}^{Site1\text{-}Ala}$
9	-	$MogR_{1\text{-}151}$

Supplementary Figure S11. *E. coli* two-hybrid interaction assay to confirm the critical roles of the site-1 and interdomain interactions in GmaR-MogR binding (n = 3 independent experiments). GmaR ($GmaR_{FL}$ or $GmaR_{FL}^{E293A}$) and MogR ($MogR_{D}$, $MogR_{1\text{-}151}$, or $MogR_{D}^{Site1\text{-}Ala}$) were fused with the T18 and T25 fragments of adenylate cyclase, respectively, and coexpressed in *E. coli* at 30 and 37°C. The β-galactosidase activity of the cells was measured to monitor GmaR-MogR binding. High β-galactosidase activity was observed for the $GmaR_{FL}$-$MogR_{D}$ pair (column 1) at 30°C but not at 37°C, indicating the temperature-dependent interaction of GmaR and MogR. However, when site-1 residues were mutated to alanine ($MogR_{D}^{Site1\text{-}Ala}$; column 2) or removed ($MogR_{1\text{-}151}$; column 3), the β-galactosidase activity was negligible both at 30 and 37°C, suggesting that site 1 is indispensable for the GmaR-MogR interaction. When the E293 residue was mutated to alanine in $GmaR_{FL}$ (column 4) to disrupt the interdomain interaction of GmaR, β-galactosidase activity decreased at 30°C. This observation suggests that the interdomain interaction is required for GmaR to mediate its MogR-binding function.

Supplementary Figure S12

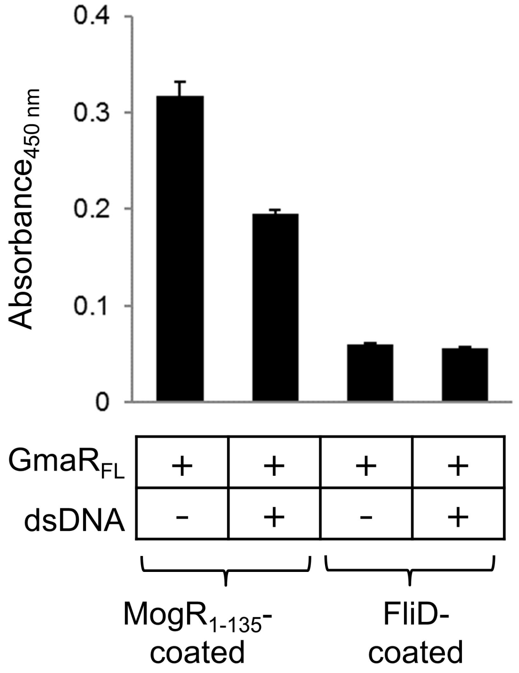

Supplementary Figure S12. Inhibition of the $MogR_{1\text{-}135}$-$GmaR_{FL}$ interaction by the operator dsDNA. The interaction of $MogR_{1\text{-}135}$ (or the negative control FliD) and $GmaR_{FL}$ was analyzed in the absence or presence of the operator dsDNA by ELISA. The data are representative of three independent experiments that yielded similar results.

Supplementary Figure S13

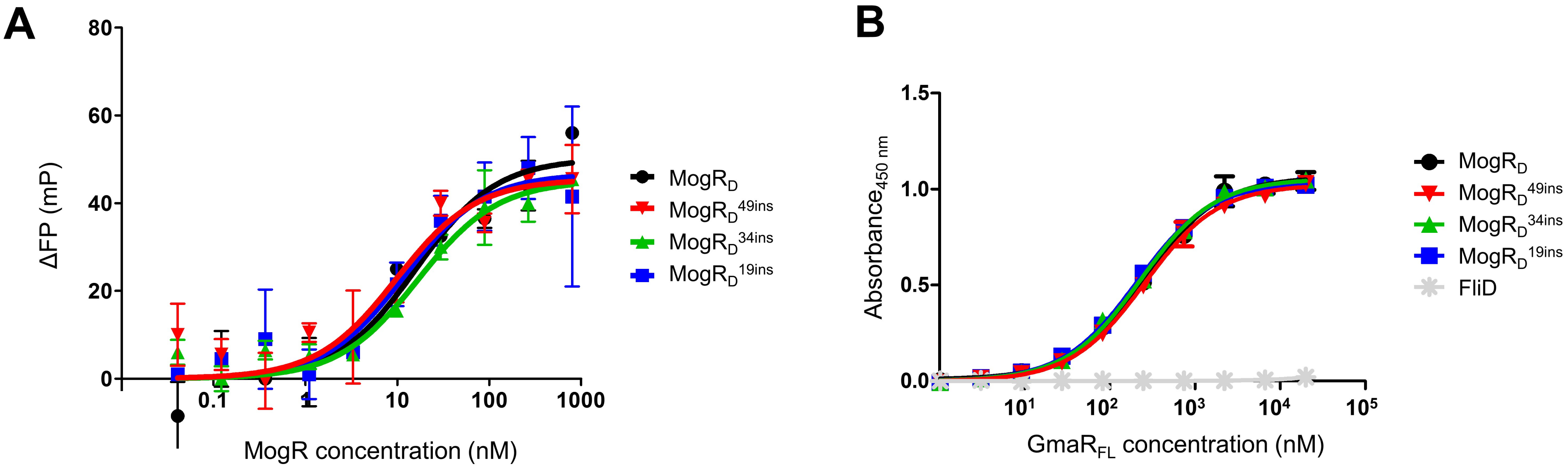

Supplementary Figure S13. Insignificant effects of insertional mutations on the dsDNA-binding and GmaR$_{FL}$-binding affinities of MogR$_D$. (**A**) No significant change in MogR$_D$-dsDNA binding by the insertional mutations. The dsDNA-binding affinities of MogR$_D$ and its insertional mutants (MogR$_D$^{49ins}, MogR$_D$^{34ins}, and MogR$_D$^{19ins}) were determined using the FP assay (n = 3 independent experiments). (**B**) No significant change in GmaR$_{FL}$-MogR$_D$ binding by the insertional mutations. The GmaR$_{FL}$-binding affinities of MogR$_D$ and its insertional mutants (MogR$_D$^{49ins}, MogR$_D$^{34ins}, and MogR$_D$^{19ins}) were determined using the ELISA (n = 4 independent experiments).

Supplementary Figure S14

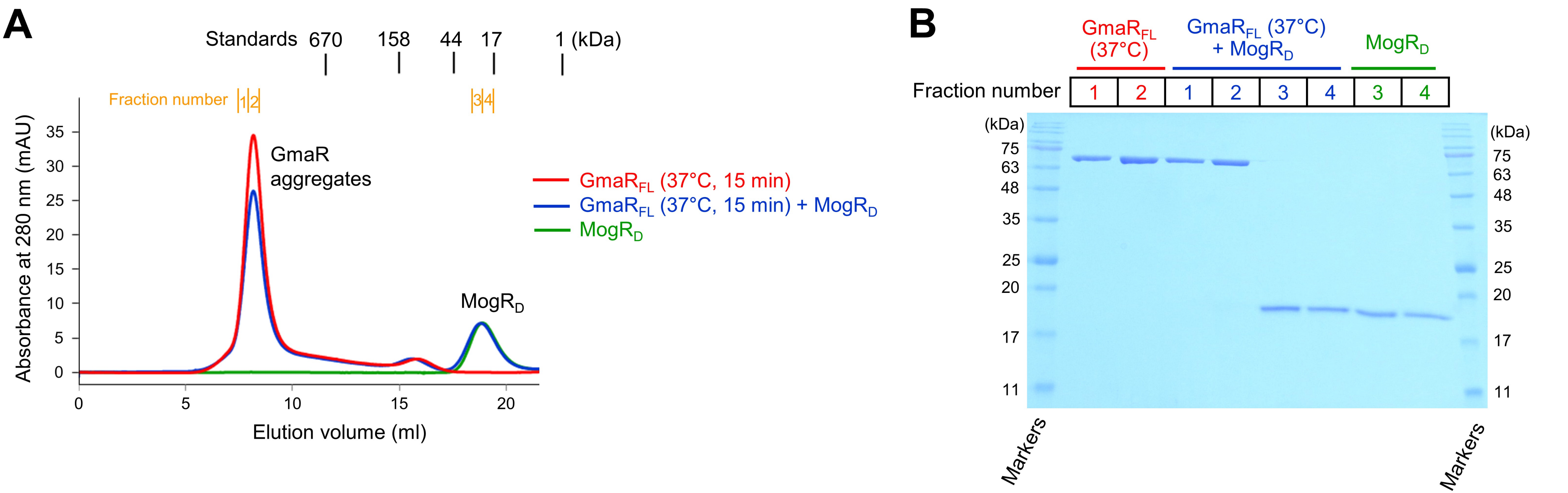

Supplementary Figure S14. Loss of the MogR-binding ability of GmaR at 37°C. The GmaR$_{FL}$ protein was preincubated at 37°C for 15 min, and the MogR$_D$-binding activity of the resulting GmaR$_{FL}$ protein was analyzed by gel-filtration chromatography (A). Fractions 1-4 obtained by gel-filtration chromatography were analyzed with SDS-PAGE (B). The data are representative of three independent experiments that yielded similar results.

Supplementary Figure S15

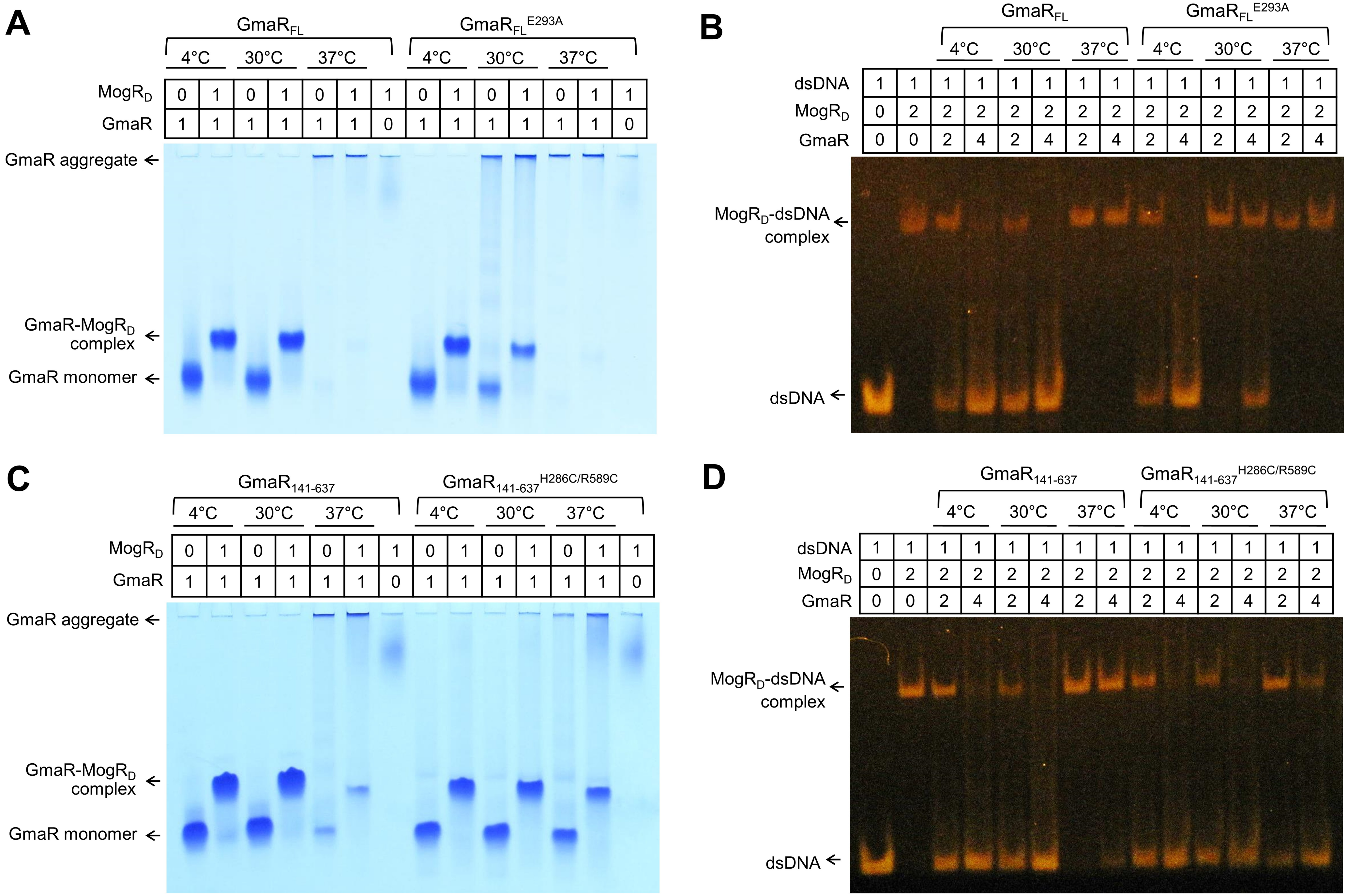

Supplementary Figure S15. Modulation of temperature-dependent GmaR functions via disruption or stabilization of the interdomain interaction. (**A**) MogR$_D$ binding to GmaR$_{FL}$ and GmaR$_{FL}^{E293A}$ proteins exposed to different temperatures. The GmaR$_{FL}$ and GmaR$_{FL}^{E293A}$ proteins were preincubated at 4, 30, or 37°C for 15 min. The resulting GmaR protein was subsequently incubated with MogR$_D$, and then the formation of GmaR aggregates and the GmaR-MogR complex was analyzed by native PAGE (n = 3 independent experiments). (**B**) Inhibition of the MogR$_D$-dsDNA interaction by the GmaR$_{FL}$ and GmaR$_{FL}^{E293A}$ proteins exposed to different temperatures. The GmaR$_{FL}$ and GmaR$_{FL}^{E293A}$ proteins were preincubated at 4, 30, or 37°C for 15 min. The resulting GmaR protein was further incubated with the 2:1 MogR$_D$:dsDNA mixture, and then the inhibitory effect of GmaR on the MogR$_D$-dsDNA interaction was determined using EMSA (n = 3 independent experiments). (**C**) MogR$_D$ binding to the GmaR$_{141-637}$ and GmaR$_{141-637}^{H286C/R589C}$ proteins exposed to different temperatures. The GmaR$_{141-637}$ and GmaR$_{141-637}^{H286C/R589C}$ proteins were pretreated at 4, 30, or 37°C for 5 min. The resulting GmaR protein was incubated with MogR$_D$, and then the formation of GmaR aggregates and the GmaR-MogR complex was analyzed using native PAGE (n = 4 independent experiments). (**D**) Inhibition of the MogR$_D$-dsDNA interaction by the GmaR$_{141-637}$ and GmaR$_{141-637}^{H286C/R589C}$ proteins exposed to different temperatures. The GmaR$_{141-637}$ and GmaR$_{141-637}^{H286C/R589C}$ proteins were preincubated at 4, 30, or 37°C for 5 min. The resulting GmaR protein was further incubated with the 2:1 MogR$_D$:dsDNA mixture, and then the inhibitory effect of GmaR on the MogR$_D$-dsDNA interaction was monitored using EMSA (n = 3 independent experiments).

Supplementary Table S1. Data collection statistics for the SeMet-GmaR^{Apo} crystal used to determine the GmaR^{Apo} structure by SAD phasing.

	SeMet-GmaR^{Apo}
**Data collection**	
Space group	$P2_12_12_1$
Cell parameters	a = 74.97 Å
	b = 85.20 Å
	c = 104.61 Å
Wavelength (Å)	0.9791
Resolution (Å)	30.00 - 2.30
Highest resolution (Å)	2.34 - 2.30
No. unique reflections	30,615 (1,504) [a]
R_{merge} (%) [b]	12.9 (52.7) [a]
I/sigma(I)	27.4 (6.1) [a]
Completeness (%)	100.0 (100.0) [a]
Redundancy	9.5 (9.8) [a]

[a] Numbers in parenthesis were calculated from data of the highest resolution shell.
[b] $R_{merge} = \Sigma_{hkl}\Sigma_i | I_i(hkl) - \langle I(hkl)\rangle | / \Sigma_{hkl}\Sigma_i I_i(hkl)$

Supplementary Table S2. Crystallographic statistics of the $GmaR^{Apo}$ and GmaR-MogR structures.

	$GmaR^{Apo}$	GmaR-MogR complex
**Data collection**		
Space group	$P2_12_12_1$	$P2_12_12_1$
Cell parameters		
a (Å)	74.74	91.87
b (Å)	85.78	91.99
c (Å)	104.31	268.99
Wavelength (Å)	1.0000	0.9794
Resolution (Å)	30.00 - 2.25	30.00 - 3.11
Highest resolution (Å)	2.29 - 2.25	3.16 - 3.11
No. unique reflections	32,149 (1,542) [a]	43,043 (2,094) [a]
R_{merge} (%) [b]	9.6 (120.4) [a]	12.1 (152.5) [a]
R_{meas} (%) [c]	11.2 (142.2) [a]	13.7 (173.7) [a]
$CC_{1/2}$ [d]	0.991 (0.495) [a]	0.993 (0.344) [a]
I/sigma(I)	19.7 (1.3) [a]	16.7 (1.3) [a]
Completeness (%)	99.3 (97.5) [a]	99.9 (100.0) [a]
Redundancy	3.7 (3.4) [a]	4.5 (4.5) [a]
**Refinement**		
Resolution (Å)	30.00 - 2.25	30.00 - 3.11
No. reflections (work)	30,430	39,674
No. reflections (test)	1,660	2,127
R_{work} (%) [e]	22.4	19.5
R_{free} (%) [f]	26.1	24.1
No. atoms		
Protein	3,596	12,093
Water	52	0
Average B-value ($Å^2$)	51.9	87.5
RMSD bonds (Å)	0.005	0.007
RMSD angles (°)	0.614	0.888
Ramachandran [g] (favored)	97.1%	97.2%
(outliers)	0.0%	0.0%

[a] Numbers in parenthesis were calculated from data of the highest resolution shell.

[b] $R_{merge} = \Sigma_{hkl}\Sigma_i | I_i(hkl) - \langle I(hkl)\rangle | / \Sigma_{hkl}\Sigma_i I_i(hkl)$

[c] $R_{meas} = \Sigma_{hkl}\{N(hkl)/[N(hkl) - 1]\}^{1/2} \Sigma_i | I_i(hkl) - \langle I(hkl)\rangle | / \Sigma_{hkl}\Sigma_i I_i(hkl)$

[d] Correlation coefficient between intensities from random half-data sets.

[e] $R_{work} = \Sigma| |F_{obs}|-|F_{calc}| | / \Sigma|F_{obs}|$, where F_{calc} and F_{obs} are the calculated and observed structure factor amplitudes, respectively.

[f] R_{free} = as for R_{work}, except that 5% of the total reflections were selected at random and omitted from refinement.

[g] Calculated using MolProbity (http://molprobity.biochem.duke.edu).
